# Large inter- and intra-case variability of first generation tau PET ligand binding in neurodegenerative dementias

**DOI:** 10.1186/s40478-018-0535-z

**Published:** 2018-05-01

**Authors:** Melissa C. Wren, Tammaryn Lashley, Erik Årstad, Kerstin Sander

**Affiliations:** 1 Institute of Nuclear Medicine and Department of Chemistry, University College London, London, UK; 2 Institute of Neurology, Department of Molecular Neuroscience, Queen Square Brain Bank for Neurological Disorders, University College London, London, UK; 3 Radiochemistry, University College London, Kathleen Lonsdale Building, 5 Gower Place, London, WC1E 6BS UK

**Keywords:** Flortaucipir, THK, Positron emission tomography, Alzheimer’s disease, Tauopathy

## Abstract

Imaging of pathological tau with positron emission tomography (PET) has the potential to allow early diagnosis of the dementias and monitoring of disease progression, including assessment of therapeutic interventions, in vivo. The first generation of tau PET tracers, including the carbazole flortaucipir and the 2-arylquinolines of the THK series, are now used in clinical research; however, concerns have been raised about off-target binding and low sensitivity.

With the aim to determine the nature of tau pathology depicted by structurally distinct tau ligands we carried out a microscopic neuropathological evaluation in *post-mortem* human brain tissue of cases with primary and secondary tauopathies. Carbazole and 2-arylquinoline binding was only observed in cases with Alzheimer’s disease and one case with frontotemporal dementia and parkinsonism linked to chromosome 17 exhibiting a R406W *MAPT* mutation. In end stage Alzheimer’s disease cases, fluorescent imaging with the carbazole T726 and the 2-arylquinoline THK-5117 revealed high inter- and intra-case variability of tracer binding, and this was corroborated by quantitative phosphorimaging with the PET tracer [^18^F]THK-5117. Microscopic analysis of the pathological inclusions revealed that the fluorescent tracers preferentially bind to premature tau aggregates. Whilst T726 binding was limited to neuronal tau, THK-5117 additionally depicted neuritic tau. Neither tracer depicted tau in pre-symptomatic disease.

Our results highlight limitations of the first generation of tau PET tracers, in particular lack of correlation between pathological tau load and tracer binding, limited sensitivity to tau in early disease, and high variability in tracer binding between and within cases. Concerns remain that these limitations may also affect the next generation tracers as they target the same high affinity binding site. Therefore, it is crucial to assess inter- and intra-subject correlation of tracer binding with pathological tau load in *post-mortem* tissue studies, and to rigorously assess novel tau PET tracers before translation into clinical studies.

**Electronic supplementary material:**

The online version of this article (10.1186/s40478-018-0535-z) contains supplementary material, which is available to authorized users.

## Introduction

Dementia represents one of the most pressing public health challenges worldwide. A clinical diagnosis of the underlying disease causing the dementia remains challenging, particularly at the early stages, and a definite confirmation is usually only obtained *post-mortem* through histopathological examination of the brain.

Approximately 80% of dementia cases are associated with structural changes of the microtubule associated protein tau (*MAPT*), causing defective tau to aggregate in neurons and glial cells [48]. The shape and distribution of these inclusions are characteristic for the respective subtypes of tauopathy. In Alzheimer’s disease, the most common dementia (50–70% of all clinical diagnoses), pathological tau load correlates closely with cognitive decline, suggesting that tau is a suitable biomarker for monitoring of disease progression [1, 14]. Quantitative assessment of tau pathology in vivo would have significant implications for medical research and for clinical practice, as it could potentially improve clinical diagnosis, predict disease progression, facilitate patient stratification and provide an outcome measure for putative disease-modifying interventions.

The first generation of tau selective ligands for non-invasive imaging with positron emission tomography (PET) have recently entered clinical studies. The clinically most advanced compound [^18^F]flortaucipir (formerly known as [^18^F]AV-1451 or [^18^F]T807), alongside the derivative [^18^F]T808 and the fluorescent analogue T726, belong to the structural class of carbazoles, whereas the different compounds of the THK series ([^18^F]THK-5105, [^18^F]THK-5117 and [^18^F]THK-5351) contain a 2-arylquinoline core. In patient studies, PET tracers from both compound classes have been reported to be suitable for staging of Alzheimer’s disease [30, 39, 40], and for differential diagnosis of Alzheimer’s disease and progressive supranuclear palsy [33, 47]. Some of these findings, in particular tracer binding to primary tauopathies, remain controversial, and are not supported by ex vivo studies on *post-mortem* brain tissue [13, 16, 22, 23, 29, 31, 37]. Furthermore, the first generation of tau PET tracers suffer from off-target binding, and in patient populations, where a signal is obtained, the sensitivity may be low [3, 36]. Efforts to develop and evaluate the next generation of tau selective tracers are ongoing [9, 15]. Pilot PET imaging studies with recently reported compounds, such as RO6958948, MK-6240 and PI-2620, suggest that these may have improved sensitivity and specificity for monitoring tau pathology in vivo. Yet, some, if not all, of the second generation tau ligands disclosed to-date display close structural similarities, and have an overlapping binding site with the first generation tau tracers. This highlights the importance of elucidating the interplay between the T808 binding site [50] and tau pathology.

We previously assessed binding of [^18^F]flortaucipir, a structural analogue of T808, to human *post-mortem* brain tissue from cases with Alzheimer’s disease and primary tauopathies, and observed a lack of correlation between PET tracer binding and total pathological tau load in the frontal and temporal cortices [37]. We also found evidence for tracer binding to a non-tau binding site. It has now been confirmed that flortaucipir exhibits strong off-target binding to monoamine oxidase isoforms and pigment containing cells, predominantly expressed in the striatum and the substantia nigra, respectively [15, 23, 46]. A similar off-target binding profile has been demonstrated for the compounds of the THK series [28, 31]. However, this does not fully explain the discrepancy between tracer binding and total tau load in the cortical brain areas.

The macroscopically observed lack of correlation between [^18^F]flortaucipir binding and total tau load [37] prompted us to investigate what tau tracers depict on a cellular level. With the aim of determining whether this discrepancy can be attributed to the binding properties of specific compounds, different compound classes, or to the tau binding site(s), we carried out a microscopic neuropathological evaluation in *post-mortem* human brain tissue of cases with Alzheimer’s disease, a range of primary tauopathies and non-demented controls with and without tau pathology. We used a combination of histology and nuclear imaging techniques to reveal ligand–tau interaction in a qualitative as well as in a quantitative manner. In particular, we aimed to answer the following questions: 1) what is the nature of tau pathology that the first generation tau ligands depict in vivo; 2) do carbazoles and 2-arylquinolines differ in their binding profiles; 3) is the T808 binding site an appropriate target for the development of tau PET tracers?

## Materials and methods

### Experimental design

Tau selective ligands were chosen to cover the most prominent compound classes of the first generation of tau PET tracers. We included the carbazoles flortaucipir and T726 as well as the 2-arylquinoline THK-5117, which is inherently fluorescent. As flortaucipir is not inherently fluorescent, we used the fluorescent structural analogue T726. T726 was used for screening purposes in the development process of flortaucipir and was shown to target the same tau binding site as flortaucipir [49]. We carried out a histopathological evaluation in human *post-mortem* brain tissue from neuropathologically well-characterised cases, selected to cover a range of tauopathies as well as controls (cf. ‘Case selection’ below). In order to assess the nature of tau pathology that the different compound classes depict, we used fluorescence microscopy with T726 and THK-5117 in conjunction with immunofluorescence. Where further clarification of tau ligand binding was required (THK-5117 binding to plaque like structures; *vide infra*), high resolution autoradiography in conjunction with immunohistochemistry was carried out. Due to the high concentration of fluorescent tau ligands needed for imaging experiments, we corroborated our results using quantitative phosphorimaging. PET tracer [^18^F]THK-5117 binding was carried out at nanomolar concentrations that would be relevant for in vivo imaging.

### Case selection

We evaluated tissue from brains donated for research to the Queen Square Brain Bank for Neurological Disorders, Institute of Neurology, University College London. All cases had undergone standard neuropathological assessment and were diagnosed according to standard criteria. Demographic data are summarised in Table 1, and additional information on tissue processing can be found in the Additional file 1: Table S1.

**Table 1:** Demographic Data of Cases Included in the Study

Case	Sex	AAO	AAD	Brain Weight (g)	Braak Stage	Thal Phase	ABC Score
CTRL1	F	–	68	1330	0	0	A0B0C0
CTRL2	M	–	71	1480	I	0	A0B1C0
CTRL3	F	–	80	1242	II	0	A0B1C0
CTRL4	M	–	89	1448	III	3	A2B2C1
AD1	F	50	66	906	VI	5	A3B3C3
AD2	M	63	73	1269	VI	5	A3B3C3
AD3	M	52	68	1234	VI	5	A3B3C3
AD4	M	63	74	1022	VI	5	A3B3C3
FTDP1^a)^	M	55	66	1208	0	0	A0B0C0
FTDP2^b)^	M	68	74	1048	II	2	A1B1C1
FTDP3^c)^	M	59	66	1399	0	0	A0B0C0
PICK1	M	57	62	1166	III	1	A1B2C1
PICK2	M	54	64	1040	0	3	A2B0C1
PSP1	M	62	74	1580	0	2	A1B0C1
PSP2	F	60	68	1177	0	1	A1B0C1
CBD	F	62	69	1100	0	0	A0B0C0

Abbreviations: CTRL, control; AD, Alzheimer’s disease; FTDP, frontotemporal dementia with parkinsonism linked to chromosome 17; PICK, Pick’s disease; PSP, progressive supranuclear palsy; CBD, corticobasal degeneration; MAPT, microtubule associated protein tau gene locus; M/F, male/female; AAO, age at onset; AAD, age at death

Genetic variants: a) MAPT R406W; b) MAPT Δ280K; c) MAPT 10 + 16

Controls were cases without a clinical history of dementia, psychiatric or neurological diseases at the time of brain donation to the Queen Square Brain Bank. Although not showing any signs of cognitive impairment at the time of death, control cases can have a considerable burden of amyloid and tau pathology (up to Thal phase 4 and Braak & Braak stage IV, respectively). The control cases included in our study had absent pathology (CTRL1), or low levels of tau and/or amyloid pathology (Braak & Braak tau stage I–III and Thal phase 1–3; CTRL 2–4). All cases with neurodegenerative diseases had dementia in life. The selected Alzheimer’s disease cases showed a high neuropathological change (CERAD frequent neuritic plaques, Braak & Braak tau stage VI and Thal phase 5 amyloid plaque pathology) using the National Institute on Aging-Alzheimer’s Association guidelines [4, 25, 26, 44]. Differential diagnosis of the FTLD-tau cases was carried out following the criteria described by the consortium for Frontotemporal Lobar Degeneration [8]. The chosen FTDP-17 cases included exonic *MAPT* mutation variants (R406W and Δ280K), and one case with an intronic 10 + 16 *MAPT* mutation. Brain areas in the disease cases were selected due to the presence of abundant tau deposits. Frontal and temporal cortices as well as the hippocampus were examined in Alzheimer’s disease, FTDP-17 and control cases. Frontal and temporal cortices were examined in Pick’s disease, frontal cortex and cerebellum in progressive supranuclear palsy, and frontal and parietal cortex in corticobasal degeneration. The frontal area used corresponded to Brodman area 9, the temporal cortex and hippocampus were taken from the level of the lateral geniculate body. The choroid plexus was not attached to the hippocampus. Cerebellum sections contained cerebellar folia as well as white matter exhibiting the dentate nuclei. Parietal cortex samples were taken from the superior parietal lobule.

### Immunohistochemistry on flash frozen tissue sections

Immunohistochemistry staining was carried out in flash frozen tissue on sequential sections to those used for autoradiography and fluorescence imaging. Tissue sections were cryosectioned at 10 μm, mounted on superfrost microscope slides and post-fixed in 4% paraformaldehyde for 30 min. Endogenous peroxidase activity was blocked using 0.3% hydrogen peroxide in methanol (10 min, room temperature). Non-specific binding was blocked and tissue permeabilised, using 10% dried non-fat milk under addition of 0.3% Triton X-100 (30 min, room temperature). Tissue sections were incubated with either AT8 (Thermoscientific, 1:600) or Aβ-XP (Cell Signaling, 1:200) for one hour at room temperature, followed by biotinylated anti-mouse or anti-rabbit IgG (DAKO, 1:200; 30 min), respectively, and avidin-biotin complex (Vector Laboratories; 30 min). Colour was developed with 3,3′-diaminobenzidine and hydrogen peroxide. Counterstaining was carried out using Mayer’s haematoxylin.

Pathological phosphorylated tau burden was detected using AT8 immunostaining. AT8 recognises the two earliest phosphorylation sites on tau and highlights all levels of neurofibrillary pathology, including intra- and extracellular tangles, pre-tangles and neuropil threads [10]. Similarly, the amyloid antibody Aβ-XP detects several isoforms of amyloid beta, including the pathogenic species Aβ-42. Moreover, the combination of AT8 (raised in mouse) and Aβ-XP (raised in rabbit) allowed double immunofluorescence staining to be carried out [51]. Immunostained slides were scanned on an Aperio ScanScope slide scanner, producing high resolution digital histology images. Regions of interest were extracted using Aperio ImageScope software. Image quantification was performed using the analysis software packages ImageJ (version 1.48) [38] and Python (version 3.6.1). Macroinstructions processing of immunohistochemistry images was conducted firstly by random box generation of 10 squares within a dedicated region of interest, and secondly by setting thresholds for the 3,3′-diaminobenzidine chromogen. Tau load was quantified in adjacent sections to those exposed to [^18^F]THK-5117, as percentage of positively stained tissue detected in regions of interest.

### Fluorescence microscopy

Tissue cryosections (10 μm) on superfrost microscope slides were post-fixed in 4% paraformaldehyde for 30 min. Autofluorescence was largely quenched by treating the sections with sudan black (0.1% in 70% ethanol) for ten minutes, followed by washing with 30% ethanol and tris buffered saline containing 0.05% Tween 20.

#### Fluorescent tau ligand binding studies

Non-specific binding was blocked and tissue permeabilised, using 10% dried non-fat milk under addition of 0.3% Triton X-100 (30 min, room temperature) [19]. Sections were incubated with either T726 (100 μM) [49] or THK-5117 (100 μM) [29] for one hour at room temperature, in a humidified chamber and under exclusion of light. Sections incubated with T726 were mounted with DAPI-containing fluorescent mounting medium (Vector Laboratories), and sections with THK-5117 were incubated with Nissl Neurotrace 640 (Molecular Probes; 1:100 for 20 min) to highlight neuronal nuclei within tissue samples.

The use of blocking buffer (composed of milk and Triton X-100) did not affect the binding of the fluorescent small molecule tau ligands (control experiments not shown), yet it helped to reduce auto fluorescence of the basal tissue extracellular matrix. In addition, comparability of results obtained from experiments in directly adjacent tissue sections (AT8 immunohistochemistry, AT8 immunofluorescence and co-staining with a tau ligand, staining with a tau ligand alone) was ensured. Nuclei markers were selected according to their compatibility with imaging of the respective tau ligand. Whilst T726 (λ_ex_ 414 nm; λ_em_ 500 nm) was imaged in conjunction with DAPI (λ_ex_ 358 nm; λ_em_ 461 nm), the excitation maxima of DAPI and THK-5117 (λ_ex_ 362 nm; λ_em_ 510 nm) were too close to allow for separate micrographs to be taken, and therefore, the neuronal nuclei marker Nissl Neurotrace 640 (λ_ex_ 640 nm; λ_em_ 660 nm) was used.

#### Fluorescent immunohistochemical co-staining with tau tracers

Tissue sections were treated as described above. An additional blocking step before the addition of milk was used to reduce endogenous peroxidase activity, using methanol containing 0.3% hydrogen peroxide. Sections were incubated with the respective primary antibody, AT8 (Thermoscientific, 1:600), AT100 (Thermoscientific, 1:500), AT180 (Thermoscientific, 1:500), PHF1 (source: Peter Davies; 1:500) or Aβ-XP (Cell Signaling, 1:200), prior to tracer labelling. Incubation with a secondary biotinylated antibody (either rabbit anti-mouse IgG or swine anti-rabbit IgG; Dako, 1:200, 30 min) was followed by treatment with avidin-biotin complex (Vector Laboratories; 30 min). Tyramine signal amplification (red or green; 1:200) was used as a substrate for horseradish peroxidase. Sections were mounted with either DAPI-containing fluorescent mounting medium or Nissl Neurotrace 640, as described above.

#### Fluorescence imaging

Fluorescence imaging was performed on a Leica epi-fluorescent microscope. Z-stacks of images were taken with a 63× oil objective. Fluorescent images were observed within specific wavelengths for maximal excitation and emission of each fluorophore. Images were subjected to three-dimensional (3D) deconvolution with the Leica LASAF image processing software. Deconvolution parameters used the Blind Method, detecting X, Y and Z volumes, with 5 iterations of each z-stack in each fluorescent channel. Using the same software, deconvoluted images were subjected to 3D projection processing, producing the final image of tau tracer and antibody binding fluorescence, as average 2D projected images of the original z-stack. Frequencies of fluorescent tau ligands binding to protein aggregates were semi-quantitatively recorded as ‘very high’, ‘high’, ‘moderate’, ‘low’, and ‘absent’, relating to pathological tau load determined by AT8 immunohistochemistry in directly adjacent tissue sections. Morphological characteristics of tau inclusions were used to differentiate pre-tangles, neurofibrillary tangles and ghost tangles. Pre-tangles were defined by their immature, small, amorphous and loosely compacted perinuclear structure [45]. Neurofibrillary tangles were classified by their larger and more densely packed mature fibrillar structure that clearly delineated the cytosolic neuronal compartments. Ghost tangles cannot reliably be detected by fluorescence microscopy; they are distinguished from neurofibrillary tangles by their lack of a nucleus and stainable cytoplasm as depicted by haemotoxylin and eosin, or by reactivity to silver. Both of these staining methods rely on detection by light microscopy [5]. Given their specific morphological characteristics, tau aggregates could clearly be differentiated from lipofuscin pigment granules, which were observed during fluorescent tracer imaging (this is pertaining to the comparably long exposure times required for imaging of T726 and THK-5117). The lipofuscin triggered fluorescence was not taken into account during the semi-quantitative assessment of tracer binding to tau pathology.

### Synthesis of compounds and radiolabelling

Flortaucipir was synthesised in house following previously published synthetic routes [43], whilst the fluorescent analogue T726 was obtained through custom synthesis (AFChemPharm). THK-5117, and the labelling precursor THK-5119 were kindly provided by Prof N. Okamura, Department of Pharmacology, Tohoku University School of Medicine, Japan.

[^18^F]THK-5117 was prepared as described previously [29]. Briefly, fluoride-18 was dissolved in 0.5 ml of a solution of potassium carbonate (15 mM) and Kryptofix (30 mM) in acetonitrile containing 15% water. The mixture was dried under a stream of nitrogen at 90 °C, followed by azeotropic drying with two subsequent additions of acetonitrile (0.5 ml). The labelling precursor THK-5119, dissolved in dimethyl sulfoxide (2 mg in 0.5 ml), was added, and the resulting mixture stirred at 110 °C for ten minutes. Hydrochloric acid (3 M, 0.5 ml) was added, and stirring was continued for five minutes. The reaction was quenched using sodium hydroxide (1 M, 1 ml) under cooling (ice bath). The radioactive product was purified on a semi-preparative Chromolith® Performance RP-18e column (Merck Millipore; 100 × 4.6 mm) at a flow rate of 5 ml/min, using a mixture of water and methanol containing 0.5% trifluoroacetic acid as mobile phase. Stepwise isocratic elution with 10% methanol in water for five minutes, followed by 38% methanol in water for ten minutes allowed isolation of the radioactive product (retention time 13 min). Reformulation in tris buffered saline (pH 7.4, containing < 10% ethanol) was carried out on a Sep-Pak C18 Plus Light cartridge (Waters). When starting with approximately 2 GBq of fluoride-18, [^18^F]THK-5117 was obtained with a decay-corrected radiochemical yield (end of synthesis) of 48% ± 8, with a radiochemical purity of > 99%, and with a specific activity of 6.3 ± 1.2 GBq/μmol.

### Quantitative phosphorimaging

Thawed and air-dried sections (10 μm) from flash frozen tissue were post-fixed in methanol (20 min), rehydrated in tris buffered saline (30 min) and incubated with a solution of [^18^F]THK-5117 in tris buffered saline (2 MBq/ml) for 15 min (1 ml per slide). After incubation, unbound [^18^F]THK-5117 was removed by washing the sections in water (five minutes) and 50% ethanol (five minutes). Internal standards were prepared by serial dilution of the [^18^F]THK-5117 solution that was used for the incubation of the brain sections. Brain sections, and internal standards absorbed on filter paper, were left to air-dry and subsequently exposed to a phosphor screen (BAS-IP MS, GE Healthcare) overnight. Phosphorimaging was performed on a Typhoon Trio scanner (GE Healthcare), at a resolution of 50 μm.

For blocking studies, sections were rehydrated in tris buffered saline containing chlorgiline and L-deprenyl (1 μM each). Incubation with [^18^F]THK-5117 was carried out under concomitant addition of different concentrations of either non-labelled THK-5117 or flortaucipir.

Phosphor images were analysed using the software ImageJ (version 1.48) [38]. Quantification of [^18^F]THK-5117 binding in manually drawn regions of interest (mean ± standard deviation in kBq/cm^2^) was based on correlation curves generated from the internal standards.

### Nuclear emulsion autoradiography

Sections were treated with [^18^F]THK-5117 as described above (Phosphorimaging). After tracer incubation, the air-dried sections were dipped in nuclear photographic emulsion K.5 (Ilford; 1:4 dilution in water) and exposed overnight under exclusion of light. Slides were washed in phenisol developer (Ilford; 20%, four minutes), acetic acid (1%, two minutes), hypam fixative solution (Ilford; 20%, four minutes) and water. Immunohistochemistry was subsequently performed as described above. The concentration of the primary antibodies was as follows: AT8 (Thermoscientific; 1:100), Aβ-XP (Cell Signalling; 1:100) and PHF-1 (source: Peter Davies; 1:100). Immunostaining controls not subject to silver dipping were carried out on adjacent tissue sections.

Nuclear emulsion autoradiography photomicrographs were assessed for accumulation of silver clusters over entire areas of brain tissue for each case assessed, including grey and white matter.

## Results

### Case characterisation

**Fig. 1:**
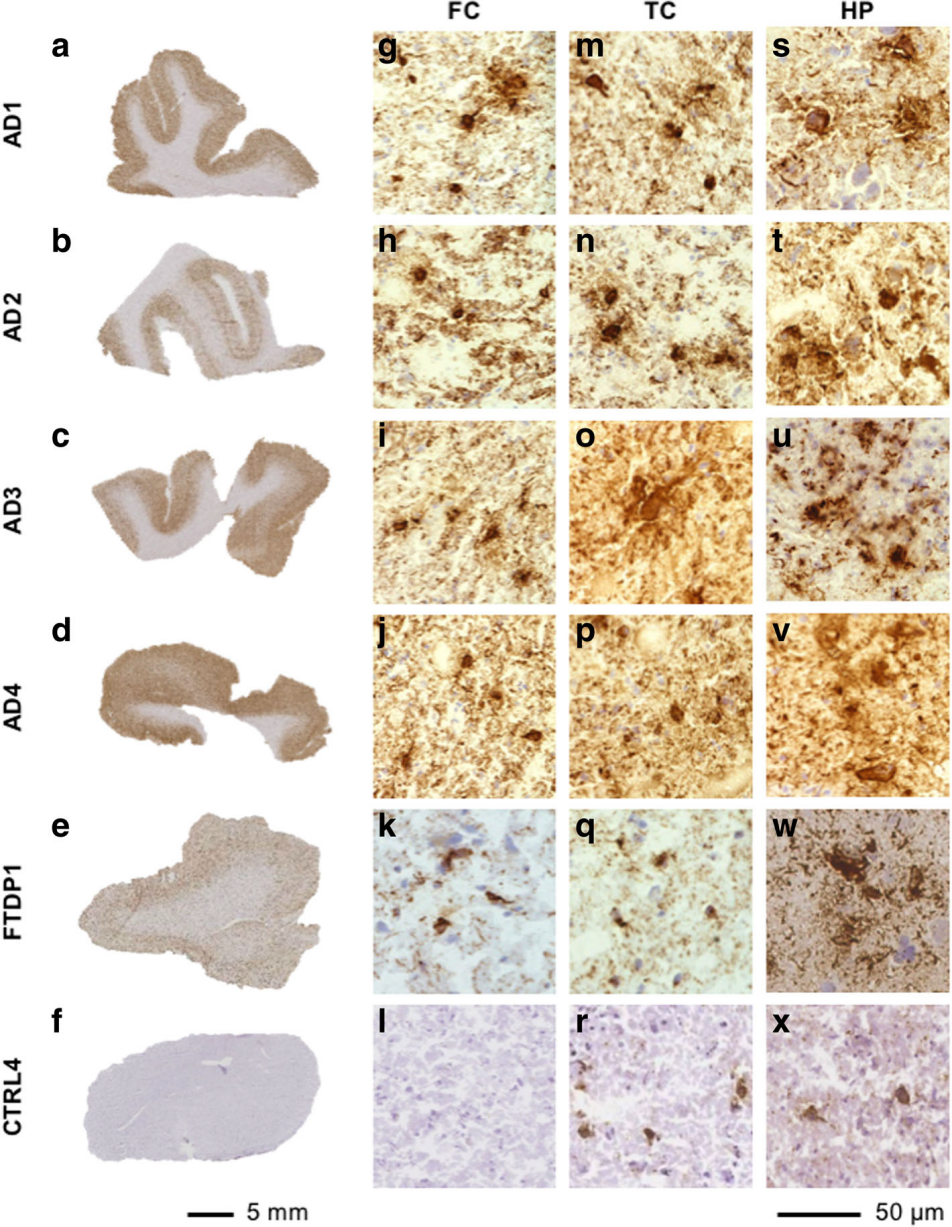
Tau immunohistochemistry in Alzheimer’s disease cases. **a**–**f**) Representative images from frontal cortex, showing macroscopic AT8 distribution in the Alzheimer’s disease cases AD1–AD4, the FTDP-17 case with a R406W *MAPT* mutation (FTDP1) and the control case CTRL4. **g**–**x**) Typical microscopic AT8 positive neurofibrillary tau aggregates in frontal cortex, temporal cortex and hippocampus. Abbreviations: AD, Alzheimer’s disease; FTDP, frontotemporal dementia with parkinsonism linked to chromosome 17; CTRL, control; FC, frontal cortex; TC, temporal cortex; HP, hippocampus

The human *post-mortem* brain tissue used for this study was characterised using immunohistochemistry with the phospho-tau selective antibody AT8. Control cases had absent (Braak & Braak stage 0), or mild tau pathology (Braak & Braak stage I–III) in the medial temporal lobe. Alzheimer’s disease cases were classified as Braak & Braak stage VI and had pronounced tau pathology in the grey matter accounting for 25–35% of the surface of the respective flash frozen tissue sections (Fig. 1). In all Alzheimer’s disease cases, dense neurofibrillary tangles, pre-tangles, as well as a meshwork of neuropil threads were observed in all brain areas investigated (Fig. 1a–d). In the FTDP-17 cases, small neurofibrillary tangles and abundant threads were identified (Fig. 1e and Additional file 1: Figure S1). Pick’s disease cases displayed Pick’s bodies in the cortical areas. Cases with progressive supranuclear palsy had pre-tangles, globose tangles, tufted astrocytes and neuropil threads in the frontal cortex and frequent coiled bodies in the cerebellum. Frequent astrocytic plaques, globose tangles and coiled bodies were identified in the case with corticobasal degeneration (Additional file 1: Figure S1).

### Microscopic assessment of the nature of pathology depicted by tau ligands

**Fig. 2:**
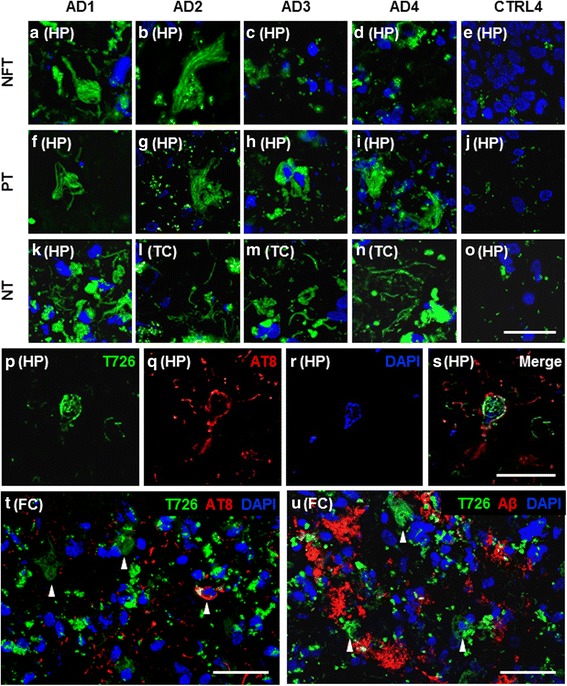
Fluorescence imaging with T726 in Alzheimer’s disease cases and controls. **a–o**) T726 bound pathology in green, nuclear marker DAPI in blue; scale bar inset 50 μm. **a–d**) T726 bound mature tangle structures in individual Alzheimer’s disease cases. **f–i**) Pre-tangle inclusions bound by T726. **k–n**) Neuropil threads bound by T726. **e/j/o**) Representative images from the control case CTRL4 depict the absence of T726 binding in control cases. **p–s**) T726 labelling of an AT8 immunoreactive hippocampal neurofibrillary tangle (Alzheimer’s disease case AD1); T726 in green, AT8 in red, DAPI in blue; scale bar inset 25 μm. **t**) Representative image of T726 labelled structures negative for AT8, and AT8 immunodecorated tangles not labelled with T726 (Alzheimer’s disease case AD1); T726 in green, AT8 in red, DAPI in blue; scale bar inset; 50 μm. **u**) Representative immunofluorescence image showing distinct binding patterns of T726 and Aβ-XP to cortical pathology (Alzheimer’s disease case AD1); T726 in green, Aβ-XP in red, DAPI in blue; scale bar inset 50 μm. Abbreviations: AD, Alzheimer’s disease; CTRL, control; NFT, neurofibrillary tangles; PT, pre-tangles; NT, neuropil threads; FC, frontal cortex; TC, temporal cortex; HP, hippocampus; Aβ, amyloid-beta

**Fig. 3:**
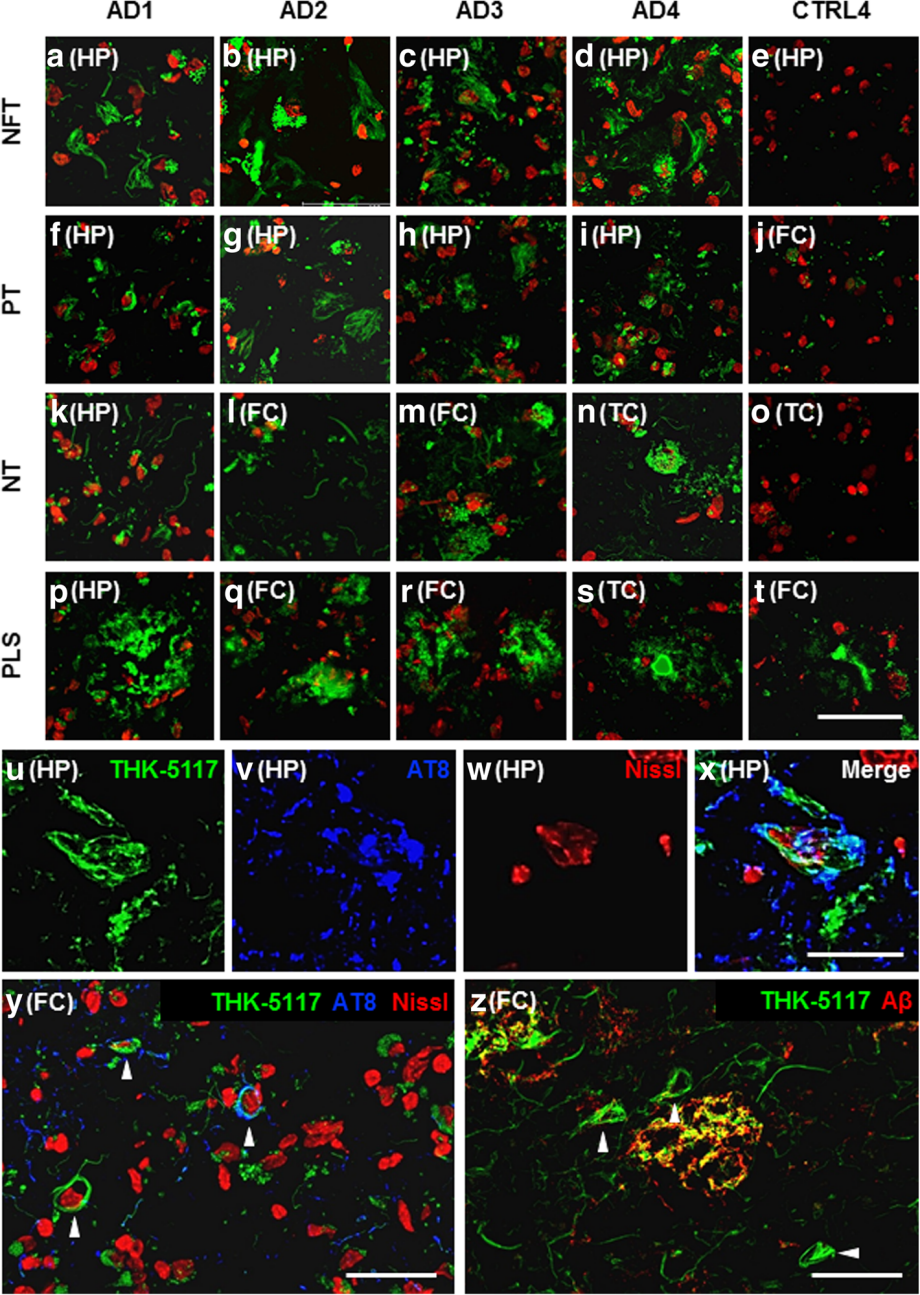
Fluorescence imaging with THK-5117 in Alzheimer’s disease cases and controls. **a–t**) THK-5117 bound pathology in green, neuronal nuclear marker, Nissl Neurotrace 640, in red; scale bar inset 50 μm. **a–d**) Images showing neurofibrillary tangle structures detected by THK-5117 in individual AD cases. **f–i**) Images that present pre-tangles bound by THK-5117. **k–n**) THK-5117 bound neuropil threads were observed only in distinct regions of each AD case. **p–s**) THK-5117 labelling of plaque-like structures in all AD cases in distinct brain regions. **e/j/o**) Representative images of THK-5117 labelling in the control case CTRL4 depict the absence of THK-5117 binding to any pathological tau structures. **t)** THK-5117 binding to plaque-like structures in control tissue. **u–x**) THK-5117 fluorescent labelling of a hippocampal neurofibrillary tangle (Alzheimer’s disease case AD1); THK-5117 in green, AT8 in blue, Nissl Neurotrace 640 in red; scale bar inset 25 μm. **y**) Representative immunofluorescence image showing THK-5117 labelled structures in absence of AT8 co-staining (Alzheimer’s disease case AD1); THK-5117 in green, AT8 in blue, Nissl Neurotrace 640 in red; scale bar inset 50 μm. **z**) Representative image of THK-5117 and Aβ-XP immunofluorescence showing distinct and co-incidental binding of THK-5117 and amyloid beta to cortical pathology; THK-5117 in green, Aβ-XP in red; scale bar inset 50 μm. Abbreviations: AD, Alzheimer’s disease; CTRL, control; NFT, neurofibrillary tangles; PT, pre-tangles; NT, neuropil threads; PLS, plaque-like structures; FC, frontal cortex; TC, temporal cortex; HP, hippocampus; Aβ, amyloid-beta

**Fig. 4:**
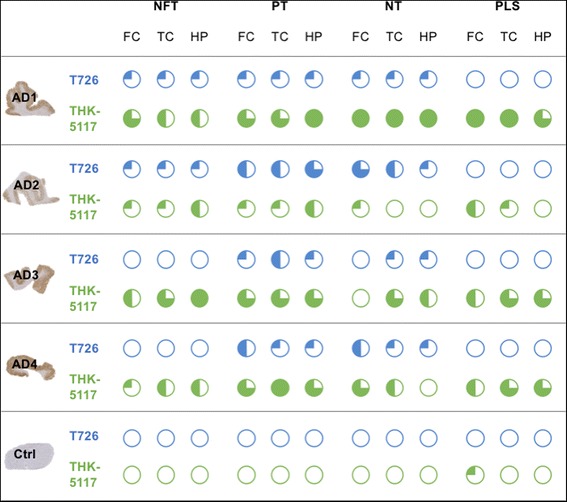
Frequency of pathology depicted by T726 (blue charts) and THK-5117 (green charts) in Alzheimer’s disease and control cases. Frequencies: , extremely frequent; , very frequent, , frequent; , infrequent; , absent; as compared to the amount of pathology depicted by the phospho-tau specific antibody AT8. Abbreviations: AD, Alzheimer’s disease; Ctrl, control cases (averaged tracer uptake in normal and pathologically aged controls); NFT, neurofibrillary tangles; PT, pre-tangles; NT, neuropil threads; PLS, plaque like structures; FC, frontal cortex; TC, temporal cortex; HP, hippocampus

**Fig. 5:**
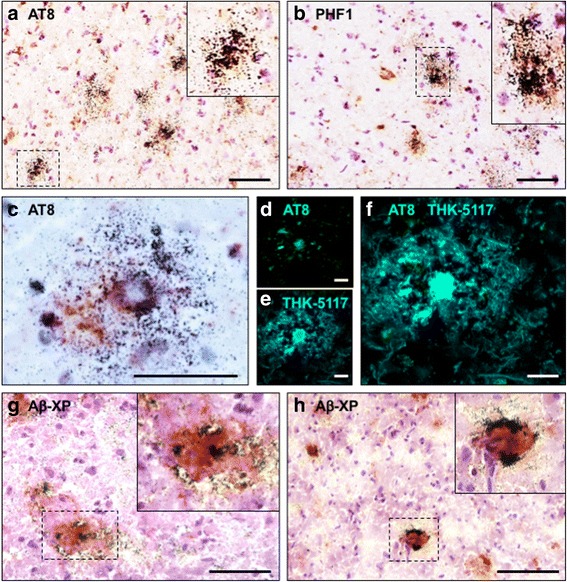
Nuclear emulsion autoradiography with [^18^F]THK-5117 in the Alzheimer’s disease case AD1. **a/b**) Silver deposition induced by [^18^F]THK-5117 binding to tau inclusions co-stained with AT8 and PHF1, respectively. **c**–**f**) Neuritic plaques labelled with [^18/19^F]THK-5117 and co-stained with AT8. **g**/**h**) Neuritic tau labelled with [^18^F]THK-5117 surrounding plaques visualised with the amyloid beta antibody Aβ-XP. Scale bar insets 100 μm

**Fig. 6:**
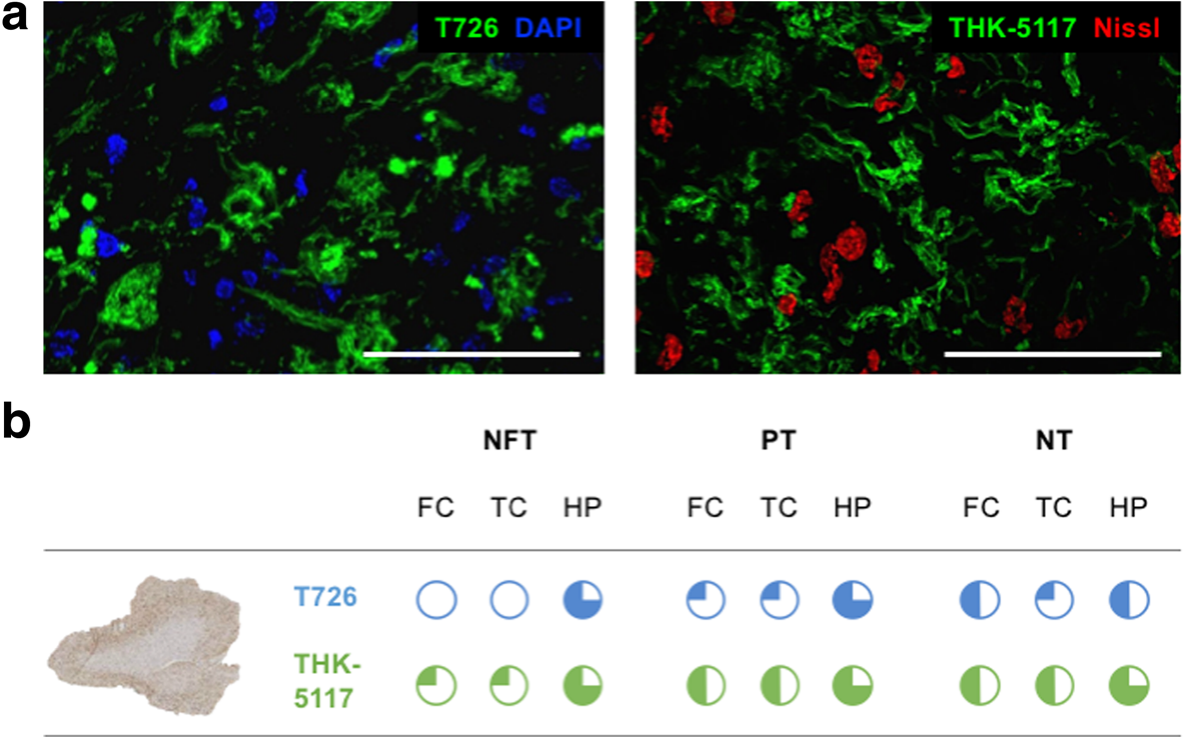
T726 and THK-5117 fluorescent labelling of the FTDP-17 case with a R406W MAPT mutation. **a)** T726 and THK-75117 binding to the CA1 section of the hippocampus; fluorescent tracers in green, DAPI in blue, Nissl Neurotrace 640 in red; scale bar inset 100 μm. **b)** Frequencies of tau pathology depicted by the fluorescent tau ligands as compared to the amount of pathology immunodecorated by the phospho-tau specific antibody AT8. Frequencies: , extremely frequent; , very frequent, , frequent; , infrequent; , absent. Abbreviations: NFT, neurofibrillary tangles; PT, pre-tangles; NT, neuropil threads; FC, frontal cortex; TC, temporal cortex; HP, hippocampus; IHC, immunohistochemistry

In order to assess the binding profiles of tau PET tracers from distinct compound classes, we carried out fluorescence microscopy with the carbazole T726 and the 2-arylquinoline THK-5117. This revealed tracer binding to tissue from cases with Alzheimer’s disease (Fig. 2–5) and the FTDP-17 case with the R406W *MAPT* mutation (Fig. 6), but not to cases with Pick’s disease, progressive supranuclear palsy, corticobasal degeneration, and FTDP-17 with Δ280K or 10 + 16 *MAPT* mutation (Additional file 1: Figure S2). No fluorescent tracer binding was observed in the control cases; although tau inclusions were confirmed in the medial temporal lobe of control cases CTRL3 and CTRL4 that had clear signs of pathological aging (A0B1C0 and A2B2C1, respectively), these were not depicted by T726 and THK-5117 (Additional file 1: Figure S3).

In the Alzheimer’s disease cases AD1–AD4, both fluorescent tau ligands depicted neurofibrillary tangles, pre-tangles and neuropil threads (Fig. 2a–o and Fig. 3a–t). On selected neurofibrillary tangles, we observed fluorescent ligand binding alongside immunoreactivity with the phospho-tau specific antibody AT8 (Fig. 2p–s, Fig. 3u–x and Additional file 1: Figure S4). Overall, the fluorescent tracers bound to fewer pathological inclusions than were depicted by antibodies: following semiquantitative assessment of the fluorescence microscopy images, T726 depicted approximately 20–30% of total pathological tau burden, as determined by AT8 immunohistochemistry, whereas THK-5117 labelled 50–80% of tau inclusions. However, both T726 and THK-5117 depicted neurons that were negative not only for the phospho-tau specific antibody AT8 (Fig. 2t and Fig. 3y), but also for antibodies detecting epitopes that are typically phosphorylated at later disease stages (AT100, AT180, PHF1) and that are therefore considered to be markers for mature neurofibrillary tangles (Additional file 1: Figure S5).

The fluorescent ligands showed high variability in depicting tau pathology across Alzheimer’s disease cases, despite all cases having reached Braak & Braak stage VI and Thal phase 5. Regional differences in tracer binding were observed, yet there was no consistency in the binding pattern of the different compound classes (Fig. 4). T726 depicted low amounts of neurofibrillary tangles, pre-tangles and neuropil threads in the hippocampus, frontal and temporal cortices of case AD1. Similarly, in the case AD2, only a low percentage of neurofibrillary tangles was detected, but moderate to high degree of tracer binding to pre-tangles and threads was observed. Surprisingly, T726 binding to mature neurofibrillary tangles was absent in the cases AD3 and AD4. Pre-tangles and neuropil threads were identified by T726 in these cases, albeit in low amounts. Using THK-5117 in the case AD1, very high amounts of neurofibrillary tangles, pre-tangles and neuropil threads were identified in the three brain areas investigated. In this case, the tau load depicted by the fluorescent tracer was comparable to that found by immunohistochemistry with AT8 in the directly adjacent brain sections. In the case AD2, THK-5117 only depicted low amounts of tau pathology, compared to those found with AT8. High, and very high, percentage of neurofibrillary tangles, pre-tangles and threads was observed throughout brain areas in the cases AD3 and AD4, respectively.

Only THK-5117 (Fig. 3z), but not T726 (Fig. 2u), visualised plaque-like structures in the Alzheimer’s disease cases. Using nuclear emulsion autoradiography with [^18^F]THK-5117, in conjunction with immunohistochemistry, the nature of these plaque-like structures was further characterised (Fig. 5). [^18^F]THK-5117 binding to neurofibrillary tangles that were immunoreactive for the phospho-tau specific antibodies AT8 and PHF1 was observed in all four Alzheimer’s disease cases (Fig. 5a/b). Additionally, silver clusters co-localised with dystrophic neurites surrounding the core of neuritic plaques, following the same distribution pattern that was detected with the fluorescent tracer (Fig. 5c–f). The dense silver deposits surrounded by amyloid, as indicated by Aβ-XP staining (Fig. 5g/h), are likely to be ghost tangles or the cores of plaques.

Both T726 and THK-5117 bound to tissue from the FTDP-17 case with the R406W *MAPT* mutation (FTDP1), but not to cases with the Δ280K or the 10 + 16 variant (FTDP2/3). In FTDP1 we observed fluorescent T726 and THK-5117 labelling of a high amount of neurofibrillary tangles, pre-tangles and neuropil threads in the hippocampus (Fig. 6a). In the cortical areas, both tracers depicted tau inclusions with a lower frequency. Whereas THK-5117 binding reflected pathological tau load as determined by AT8, T726 lacked binding to neurofibrillary tangles in the temporal and frontal cortices of this case (Fig. 6b).

### Quantitative phosphorimaging with [^18^F]THK-5117

**Fig. 7:**
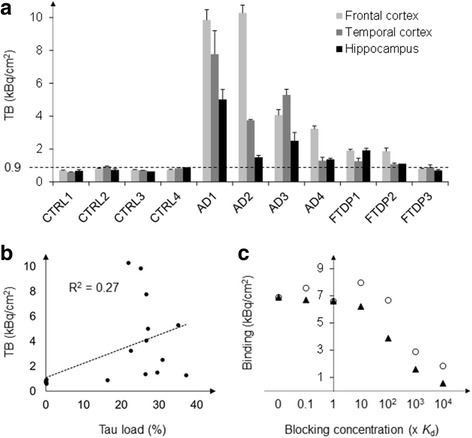
Quantitative phosphorimaging with [^18^F]THK-5117 **a**) Total binding of [^18^F]THK-5117 to the grey matter of control and tauopathy cases. Experiments were carried out in triplicates. **b**) Correlation of total [^18^F]THK-5117 binding in the grey matter, as determined by quantitative phosphorimaging, and tau total load, as determined by AT8 immunohistochemistry in adjacent brain sections. **c**) [^18^F]THK-5117 binding to the frontal cortex of case AD1 under constant inhibition of the monoamine oxidases A and B (using 1 μM chlorgylin and L-deprenyl, respectively) and with block of tracer binding using increasing concentrations of flortaucipir (circles) or THK-5117 (triangles) ranging from 0.1–10,000 times *K*_d_. Abbreviations: CTRL, control; AD, Alzheimer’s disease; FTDP, frontotemporal dementia with parkinsonism linked to chromosome 17 (microtubule-associated protein tau mutation); TB, total binding; FC, frontal cortex; TC, temporal cortex; HP, hippocampus

In order to corroborate the high inter- and intra-case variability of tau tracer binding, as observed with fluorescent imaging, we carried out quantitative phosphorimaging with [^18^F]THK-5117 in disease groups that had shown fluorescent tracer binding, i.e. Alzheimer’s disease and FTDP-17, as well as control cases (Fig. 7 and Additional file 1: Figure S6).

Total [^18^F]THK-5117 binding (Fig. 7a) in control cases with and without brain pathology was low, ranging between 0.6 and 0.9 kBq/cm^2^ in the three brain areas investigated. In particular, there was no difference in tracer binding to the medial temporal lobe of cases with and without signs of pathological aging, despite existing pathology in the hippocampus and entorhinal cortex of the control case CTRL4 (Supporting Fig. 3). The Alzheimer’s disease case 1 (AD1) showed strong [^18^F]THK-5117 binding in the grey matter of frontal and temporal cortices as well as in the hippocampus (9.9, 7.8 and 5.0 kBq/cm^2^, respectively). AD2 showed a comparably high total binding in the frontal cortex (10.3 kBq/cm^2^), but lower uptake in temporal cortex and hippocampus (3.8 and 1.5 kBq/cm^2^, respectively). Total binding in tissue sections of the case AD3 was approximately half of that observed in AD1, but still delineated well from control cases (4.1, 5.3 and 2.5 kBq/cm^2^ in frontal, temporal cortex and hippocampus, respectively). Interestingly, the case AD4 showed low [^18^F]THK-5117 binding in the frontal cortex (3.2 kBq/cm^2^), and particularly low uptake in temporal cortex and hippocampus (1.3 and 1.4 kBq/cm^2^, respectively). The correlation of total tau load, as determined by immunohistochemistry with the phospho-tau specific antibody AT8, and total [^18^F]THK-5117 binding to the grey matter of all Alzheimer’s disease and control cases was low (R^2^ of 0.27; Fig. 7b).

The FTDP-17 case harbouring the R406W mutation (FTDP1) showed slightly increased [^18^F]THK-5117 binding in both frontal cortex and hippocampus (1.9 kBq/cm^2^) when compared to control cases. FTDP2 with a 10 + 16 mutation showed similar uptake in the frontal cortex (1.9 kBq/cm^2^), whereas in the temporal cortex and the hippocampus of this case as well as in all brain areas of case FTDP3 (Δ280K mutation) total binding was comparable to that in controls (< 1.1 kBq/cm^2^).

In order to probe the specificity of the [^18^F]THK-5117 binding we carried out blocking studies to determine the degree of non-specific, as well as off-target binding, using the frontal cortex of Alzheimer’s disease case AD1 (Fig. 7c). In these experiments, both isoforms of monoamine oxidases, A and B, were blocked using chlorgiline and L-deprenyl, respectively, leading to an overall reduction of [^18^F]THK-5117 binding from 9.9 to 6.9 kBq/cm^2^ (30%). Non-specific [^18^F]THK-5117 binding was high, and could only be reduced to < 20% of total binding when the concentration of the blocking agent exceeded 1000 times the value of the dissociation constant (*K*_d_ of THK-5117 = 5.2 nM). Displacement of [^18^F]THK-5117 with flortaucipir was observed in the micromolar concentration range (40% of total [^18^F]THK-5117 binding at a blocking concentration of 1000 x *K*_d_ (AV-1451) = 14.6 nM).

## Discussion

Tau ligand binding in the Alzheimer’s disease cases used in this study did not reflect the high, and coherent, pathological tau load as determined by immunohistochemistry. Using fluorescent as well as radiolabelled tau ligands, we observed a large inter- and intra-case variability in tracer binding, and there was no dominant or consistent uptake pattern shared by the structurally different compounds. In contrast, staining with the phospho-tau specific antibody AT8 confirmed all Alzheimer’s disease cases to be affected by severe tau pathology (Braak & Braak stage VI) throughout the brain areas investigated. In life, all cases had been diagnosed with the sporadic variant, and magnetic resonance imaging had shown generalised cortical atrophy typical for late stage disease.

Total [^18^F]THK-5117 binding to the grey matter of the Alzheimer’s disease cases ranged from 1.3 to 10.3 kBq/cm^2^, and the lower uptake value barely delineated from that found in control tissue. In particular, one case (AD2) showed low frequency of tau pathology depicted by THK-5117 compared to that found in the other Alzheimer’s disease cases, whereas the cases AD3 and AD4 appeared to be low-affinity binders for T726. Using fluorescence microscopy with THK-5117 and T726, we noted that not only the frequency, but also the type of the pathological inclusions depicted by the different compounds varied. Surprisingly, T726 binding to mature neurofibrillary tangles was only observed in the cases AD1 and AD2, but not in the cases AD3 and AD4; THK-5117 depicted neurofibrillary tangles with moderate to high frequency in all cases and brain areas investigated. Both tracers seemed to preferentially depict premature tau inclusions, such as pre-tangles and neuropil threads. Apart from the case AD2, i.e. the low-affinity binder for THK-5117, pre-tangles were found consistently throughout cases and brain areas using both tracers, whilst there were particular brain areas that exhibited a low frequency of threads, such as the frontal cortex of AD3 and the hippocampus of AD4. Interestingly, the 2-arylquinoline THK-5117 showed preferential binding to neuritic tau, whereas carbazole T726 binding was limited to neuronal tau. [^18^F]THK-5117 binding was partially displaced by flortaucipir, confirming that the binding sites for the 2-arylquinoline and the carbazole overlap. Differences in the binding profile of the two compound classes may pertain to an additional binding site found for the 2-arylquinolines [6, 20].

The large variability in T726 and [^18/19^F]THK-5117 binding to tissue from Alzheimer’s disease cases is consistent with results from our previous study with [^18^F]flortaucipir, which demonstrate a lack of correlation between tau ligand binding to *post-mortem* brain tissue and pathological tau load [37]. PET imaging in patients with Alzheimer’s disease has shown [^18^F]flortaucipir binding to be closely linked to brain areas affected by neurodegeneration, and tracer uptake to correlate with the degree of cognitive impairment [17, 32, 34]. However, in some Alzheimer’s disease patients, who were positively tested for amyloid, [^18^F]flortaucipir retention in the cortical areas was found to be low, and comparable to that observed in age-matched controls. Other study participants showed highly focal tracer binding in limited cortical areas [35]. A similar variability – despite smaller patient cohorts – was observed when using PET tracers from the THK series [13, 21]. These studies support the notion that PET tracer binding is dependent on the strain of tau pathology in the respective patient [7, 18, 27] and that there may be populations expressing tau inclusions with a particularly high, or low, affinity to the first generation tau PET tracers.

[^18^F]Flortaucipir binding was recently reported to correlate with neurofibrillary tangle load, as determined by PHF1 antibody staining in *post-mortem* brain tissue from cases representing the different Braak & Braak stages (I–VI) [24]. In our hands, neither the structural analogue T726, nor the 2-arylquinoline THK-5117, depicted tau inclusions observed in the control cases with pathology (Braak & Braak stages II and III). Furthermore, the tau tracers did not exclusively bind to neurofibrillary tangles, but predominantly to premature tau inclusions such as pre-tangles and neuropil threads (*vide supra*). This is in contrast to observations made by others, which support the notion that flortaucipir [22, 31] and THK-5117 [11, 12] primarily depict mature tau inclusions. Methodological differences, in particular the use of paraffin embedded tissue that requires dewaxing and antigen retrieval methods before staining, as well as inter-case variability may explain different interpretations. Given the strong evidence of our fluorescence microscopy study for tau tracer binding to both premature and mature inclusions, we used the phospho-tau specific antibody AT8 to determine total pathological tau load in the tissue used for this study, as it detects the earliest phosphorylation sites on tau, resulting in the development of neurofibrillary structures. The low sensitivity for tau inclusions in prodromal disease cases, as reflected by the ratio of pathological tau depicted by the small molecule ligands to that depicted by AT8, may preclude detection of disease in vivo at the earliest stages. Over the course of disease, maturation of the tau inclusions may affect the accessibility of the binding site(s) for tau tracers, or the avidity of binding, for instance through gradual changes to the three-dimensional structure and density of the aggregates.

In this study, we did not observe any fluorescent tau tracer binding to inclusions predominantly composed of either three- or four-repeat tau isoforms. However, tau tracer binding was detected in the FTDP-17 case with a R406W *MAPT* mutation (case FTDP1), which produces pathological inclusions that closely resemble those found in Alzheimer’s disease. Tau load in the hippocampus of the case FTDP1, as determined by immunohistochemistry with AT8, was comparable to that found in the Alzheimer’s disease cases (ca. 30%), whereas in the cortical areas it was considerably lower (ca. 15%). This was corroborated using fluorescence microscopy and nuclear emulsion autoradiography. The results also reflect findings from [^18^F]flortaucipir PET scans in patients with *MAPT* mutations that cause tau inclusions with mixed three- and four-repeat pathology (R406W and V337 M) [41, 42]. Although overall tau ligand binding in patients with the R406W and V337 M variants, as well as in the case included in our study, was lower than observed in subjects and cases with Alzheimer’s disease, the distribution pattern differed in that tracer uptake is highly focal (hippocampus and surrounding temporal cortex) in the mutation carriers. Therefore, the first generation tau PET tracers may be suitable for monitoring disease progression in patients who carry the mentioned MAPT mutations; however, further studies are needed to clarify the specificity of this technique. In particular, contradictory findings of flortaucipir binding to patients and cases with *MAPT* mutations resulting in predominantly four-repeat pathology have been reported [2, 22, 24, 37].

In summary, our results highlight the limitations of first generation tau ligands, bearing carbazole and 2-arylquinoline core structures, for some clinical applications. In particular, the high variability of tau PET tracer binding within and between late-stage sporadic Alzheimer’s disease, and the relatively low sensitivity to tau in earliest Braak stages may limit these tracers’ use for early diagnosis, monitoring of disease progression or assessment of therapeutic interventions. These limitations may be overcome by the next generation of tau PET tracers. However, it remains a concern that several of the newer tracers also target the T808 binding site, and therefore may be affected by some of the drawbacks associated with the first generation tracers. Future studies in human *post-mortem* brain tissue are crucial to address this concern.

Our study highlights the importance of applying a range of research techniques to the non-clinical assessment of tau PET tracers. Despite the different tracer concentrations used for fluorescence and nuclear tissue imaging, the results obtained with the different techniques demonstrated the same tracer binding patterns and could therefore be used to obtain complementary information about the nature of tau pathology depicted by the structurally distinct tau ligands at the cellular level. Using this unique combination of nuclear imaging and histological methods allowed us to qualitatively and quantitatively assess tracer binding at different levels of resolution. Importantly, we were able to detect tracer binding patterns resembling those observed in clinical PET studies, in particular the high variability in tracer binding between and within cases and patients, respectively. In the future, novel PET tracers should be subject to rigorous non-clinical assessment before being translated into clinical studies.

## Additional files

Additional file 1: **Table S1.** Extended demographic data of cases included in the study. **Figure S1.** Tau immunohistochemistry in cases with primary tauopathies. **Figure S2.** Lack of fluorescent tau tracer binding in cases with primary tauopathies. **Figure S3.** Lack of tau tracer binding to the medial temporal lobe of control cases CTRL1–CTRL4 with and without tau pathology. **Figure S4.** Representative immunofluorescence images showing AT8 immunoreactive tau inclusions depicted by fluorescent tau tracers. **Figure S5.** Concomitant labelling of tissue from Alzheimer’s disease cases with fluorescent tau tracers and phospho-tau specific antibodies. **Figure S6.** Quantitative phosphorimaging with [^18^F]THK-5117. (PDF 2092 kb)

## References

[1] Arriagada PV, Growdon JH, Hedley-Whyte ET, Hyman BT (1992) Neurofibrillary tangles but not senile plaques parallel duration and severity of Alzheimer's disease. Neurology 42(3):631–639. 10.1212/WNL.42.3.631 10.1212/wnl.42.3.631 1549228

[2] Bevan Jones WRB, Cope TE, Passamonti L, Fryer TD, Hong YT, Aigbirhio F, Kril JJ, Forrest SL, Allinson K, Coles JP, Jones PS, Spillantini MG, Hodges JR, O’Brien JT, Rowe JB (2016). [^18^F]AV-1451 PET in behavioural variant frontotemporal dementia due to MAPT mutation. Ann Clin Transl Neurol.

[3] Bischof GN, Endepols H, van Eimeren T, Drzezga A (2017). Tau-imaging in neurodegeneration. Methods.

[4] Braak H, Braak E (1991). Neuropathological stageing of Alzheimer-related changes. Acta Neuropathol.

[5] Braak H, Braak E (1994). Morphological criteria for the recognition of Alzheimer's disease and the distribution pattern of cortical changes related to this disorder. Neurobiol Aging.

[6] Cai L, Qu B, Hurtle BT, Dadiboyena S, Diaz-Arrastia R, Pike VW (2016). Candidate PET radioligand development for neurofibrillary tangles: two distinct radioligand binding sites identified in postmortem Alzheimer's disease brain. ACS Chem Neurosci.

[7] Castillo-Carranza DL, Guerrero-Muñoz MJ, Sengupta U, Gerson JE (in press) Kayed R (2018) α-Synuclein oligomers induce a unique toxic tau strain. Biol Psychiatry 10.1016/j.biopsych.2017.12.018 10.1016/j.biopsych.2017.12.018 PMC6201292 29478699

[8] Dickson DW, Hauw JJ, Agid Y, Litvan I, Dickson DW, Weller RO (2011). Progressive supranuclear palsy and corticobasal degeneration. Neurodegeneration: the molecular pathology of dementia and movement disorders. Wiley-Blackwell, City.

[9] Gobbi LC, Knust H, Körner M, Honer M, Czech C, Belli S, Muri D, Edelmann MR, Hartung T, Erbsmehl I, Grall-Ulsemer S, Koblet A, Rueher M, Steiner S, Ravert HT, Mathews WB, Holt DP, Kuwabara H, Valentine H, Dannals RF, Wong DF, Borroni E (2017). Identification of three novel radiotracers for imaging aggregated tau in Alzheimer's disease with positron emission tomography. J Med Chem.

[10] Goedert M, Jakes R, Vanmechelen E (1995). Monoclonal antibody AT8 recognises tau protein phosphorylated AT both serine 202 and threonine 205. Neurosci Lett.

[11] Harada R, Okamura N, Furumoto S, Furukawa K, Ishiki A, Tomita N, Hiraoka K, Watanuki S, Shidahara M, Miyake M, Ishikawa Y, Matsuda R, Inami A, Yoshikawa T, Tago T, Funaki Y, Iwata R, Tashiro M, Yanai K, Arai H, Kudo Y (2015). [(18)F]THK-5117 PET for assessing neurofibrillary pathology in Alzheimer's disease. Eur J Nucl Med Mol Imaging.

[12] Harada R, Okamura N, Furumoto S, Furukawa K, Ishiki A, Tomita N, Tago T, Hiraoka K, Watanuki S, Shidahara M, Miyake M, Ishikawa Y, Matsuda R, Inami A, Yoshikawa T, Funaki Y, Iwata R, Tashiro M, Yanai K, Arai H, Kudo Y (2016). ^18^F-THK5351: a novel PET radiotracer for imaging neurofibrillary pathology in Alzheimer disease. J Nucl Med.

[13] Harada R, Okamura N, Furumoto S, Tago T, Yanai K, Arai H, Kudo Y (2016). Characteristics of tau and its ligands in PET imaging. Biomol Ther.

[14] Haroutunian V, Davies P, Vianna C, Buxbaum JD, Purohit DP (2007). Tau protein abnormalities associated with the progression of Alzheimer disease type dementia. Neurobiol Aging.

[15] Hostetler ED, Walji AM, Zeng Z, Miller P, Bennacef I, Salinas C, Connolly B, Gantert L, Haley H, Holahan M, Purcell M, Riffel K, Lohith TG, Coleman P, Soriano A, Ogawa A, Xu S, Zhang X, Joshi E, Della Rocca J, Hesk D, Schenk DJ, Evelhoch JL (2016). Preclinical characterization of ^18^F-MK-6240, a promising PET tracer for *in vivo* quantification of human neurofibrillary tangles. J Nucl Med.

[16] Ishiki A, Harada R, Okamura N, Tomita N, Rowe CC, Villemagne VL, Yanai K, Kudo Y, Arai H, Furumoto S, Tashiro M, Furukawa K (2017). Tau imaging with [^18^F]THK-5351 in progressive supranuclear palsy. Eur J Neurol.

[17] Johnson KA, Schultz A, Betensky RA, Becker JA, Sepulcre J, Rentz D, Mormino E, Chhatwal J, Amariglio R, Papp K, Marshall G, Albers M, Mauro S, Pepin L, Alverio J, Judge K, Philiossaint M, Shoup T, Yokell D, Dickerson B, Gomez-Isla T, Hyman B, Vasdev N, Sperling R (2016). Tau positron emission tomographic imaging in aging and early Alzheimer disease. Ann Neurol.

[18] Kaufman SK, Sanders DW, Thomas TL, Ruchinskas AJ, Vaquer-Alicea J, Sharma AM, Miller TM, Diamond MI (2016). Tau prion strains dictate patterns of cell pathology, progression rate, and regional vulnerability in vivo. Neuron.

[19] Lashley T, Rohrer JD, Bandopadhyay R, Fry C, Ahmed Z, Isaacs AM, Brelstaff JH, Borroni B, Warren JD, Troakes C, King A, Al-Saraj S, Newcombe J, Quinn N, Ostergaard K, Schrøder HD, Bojsen-Møller M, Braendgaard H, Fox NC, Rossor MN, Lees AJ, Holton JL, Revesz T (2011). A comparative clinical, pathological, biochemical and genetic study of fused in sarcoma proteinopathies. Brain.

[20] Lemoine L, Saint-Aubert L, Marutle A, Antoni G, Eriksson JP, Ghetti B, Okamura N, Nennesmo I, Gillberg PG, Nordberg A (2015) Visualization of regional tau deposits using ^3^H-THK5117 in Alzheimer brain tissue. Acta Neuropathol Commun 3(40) 10.1186/s40478-015-0220-4 10.1186/s40478-015-0220-4 PMC4489196 26134112

[21] Lockhart SN, Baker SL, Okamura N, Furukawa K, Ishiki A, Furumoto S, Tashiro M, Yanai K, Arai H, Kudo Y, Harada R, Tomita N, Hiraoka K, Watanuki S, Jagust WJ (2016). Dynamic PET measures of tau accumulation in cognitively normal older adults and Alzheimer's disease patients measured using [^18^F]THK-5351. PLoS One.

[22] Lowe VJ, Curran G, Fang P, Liesinger AM, Josephs KA, Parisi JE, Kantarci K, Boeve BF, Pandey MK, Bruinsma T, Knopman DS, Jones DT, Petrucelli L, Cook CN, Graff-Radford NR, Dickson DW, Petersen RC, Jack CR, Murray ME (2016). An autoradiographic evaluation of AV-1451 tau PET in dementia. Acta Neuropathol Commun.

[23] Marquié M, Normandin MD, Vanderburg CR, Costantino IM, Bien EA, Rycyna LG, Klunk WE, Mathis CA, Ikonomovic MD, Debnath ML, Vasdev N, Dickerson BC, Gomperts SN, Growdon JH, Johnson KA, Frosch MP, Hyman BT, Gómez-Isla T (2015). Validating novel tau positron emission tomography tracer [F-18]-AV-1451 (T807) on postmortem brain tissue. Ann Neurol.

[24] Marquié M, Normandin MD, Meltzer AC, Siao Tick Chong M, Andrea NV, Antón-Fernández A, Klunk WE, Mathis CA, Ikonomovic MD, Debnath M, Bien EA, Vanderburg CR, Costantino I, Makaretz S, DeVos SL, Oakley DH, Gomperts SN, Growdon JH, Domoto-Reilly K, Lucente D, Dickerson BC, Frosch MP, Hyman BT, Johnson KA, Gómez-Isla T (2017). Pathological correlations of [F-18]-AV-1451 imaging in non-alzheimer tauopathies. Ann Neurol.

[25] Mirra SS, Heyman A, McKeel D, Sumi SM, Crain BJ, Brownlee LM, Vogel FS, Hughes JP, van Belle G, Berg L, neurologists p CERAD (1991). The consortium to establish a registry for Alzheimer's disease (CERAD). Part II. Standardization of the neuropathologic assessment of Alzheimer's disease. Neurology.

[26] Montine TJ, Phelps CH, Beach TG, Bigio EH, Cairns NJ, Dickson DW, Duyckaerts C, Frosch MP, Masliah E, Mirra SS, Nelson PT, Schneider JA, Thal DR, Trojanowski JQ, Vinters HV, Hyman BT, National Institute on Aging; Alzheimer’s Association (2012). National Institute on Aging-Alzheimer's Association guidelines for the neuropathologic assessment of Alzheimer's disease: a practical approach. Acta Neuropathol.

[27] Narasimhan S, Guo JL, Changolkar L, Stieber A, McBride JD, Silva LV, He Z, Zhang B, Gathagan RJ, Trojanowski JQ, Lee VMY (2017). Pathological tau strains from human brains recapitulate the diversity of tauopathies in nontransgenic mouse brain. J Neurosci.

[28] Ng KP, Pascoal TA, Mathotaarachchi S, Therriault J, Kang MS, Shin M, Guiot MC, Guo Q, Harada R, Comley RA, Massarweh G, Soucy JP, Okamura N, Gauthier S, Rosa-Neto P (2017). Monoamine oxidase B inhibitor, selegiline, reduces ^18^F-THK5351 uptake in the human brain. Alzheimers Res Ther.

[29] Okamura N, Furumoto S, Harada R, Tago T, Yoshikawa T, Fodero-Tavoletti M, Mulligan RS, Villemagne VL, Akatsu H, Yamamoto T, Arai H, Iwata R, Yanai K, Kudo Y (2013). Novel ^18^F-labeled arylquinoline derivatives for noninvasive imaging of tau pathology in Alzheimer disease. J Nucl Med.

[30] Okamura N, Harada R, Furumoto S, Arai H, Yanai K, Kudo Y (2014). Tau PET imaging in Alzheimer's disease. Curr Neurol Neurosci Rep.

[31] Ono M, Sahara N, Kumata K, Ji B, Ni R, Koga S, Dickson DW, Trojanowski JQ, Lee VM, Yoshida M, Hozumi I, Yoshiyama Y, van Swieten JC, Nordberg A, Suhara T, Zhang MR, Higuchi M (2017). Distinct binding of PET ligands PBB3 and AV-1451 to tau fibril strains in neurodegenerative tauopathies. Brain.

[32] Ossenkoppele R, Schonhaut DR, Schöll M, Lockhart SN, Ayakta N, Baker SL, O'Neil JP, Janabi M, Lazaris A, Cantwell A, Vogel J, Santos M, Miller ZA, Bettcher BM, Vossel KA, Kramer JH, Gorno-Tempini ML, Miller BL, Jagust WJ, Rabinovici GD (2016). Tau PET patterns mirror clinical and neuroanatomical variability in Alzheimer's disease. Brain.

[33] Passamonti L, Vázquez Rodríguez P, Hong YT, Allinson KS, Williamson D, Borchert RJ, Sami S, Cope TE, Bevan-Jones WR, Jones PS, Arnold R, Surendranathan A, Mak E, Su L, Fryer TD, Aigbirhio FI, O'Brien JT, Rowe JB (2017). ^18^F-AV-1451 positron emission tomography in Alzheimer's disease and progressive supranuclear palsy. Brain.

[34] Phillips JS, Das SR, McMillan CT, Irwin DJ, Roll EE, Da Re F, Nasrallah IM, Wolk DA, Grossman M (2017) Tau PET imaging predicts cognition in atypical variants of Alzheimer's disease. Hum Brain Mapp:1–18 10.1002/hbm.23874 10.1002/hbm.23874 PMC5764792 29105977

[35] Pontecorvo MJ, Devous MD Sr, Navitsky M, Lu M, Salloway S, Schaerf FW, Jennings D, Arora AK, McGeehan A, Lim NC, Xiong H, Joshi AD, Siderowf A, Mintun MA; ^18^F-AV-1451-A05 investigators (2017) Relationships between flortaucipir PET tau binding and amyloid burden, clinical diagnosis, age and cognition. Brain 140 (3): 748–763. 10.1093/brain/aww334 10.1093/brain/aww334 PMC5382945 28077397

[36] Saint-Aubert L, Lemoine L, Chiotis K, Leuzy A, Rodriguez-Vieitez E, Nordberg A (2017). Tau PET imaging: present and future directions. Mol Neurodegener.

[37] Sander K, Lashley T, Gami P, Gendron T, Lythgoe MF, Rohrer JD, Schott JM, Revesz T, Fox NC, Årstad E (2016). Characterization of tau positron emission tomography tracer [^18^F]AV-1451 binding to postmortem tissue in Alzheimer's disease, primary tauopathies, and other dementias. Alzheimers Dement.

[38] Schneider CA, Rasband WS, Eliceiri KW (2012). NIH image to ImageJ: 25 years of image analysis. Nat Meth.

[39] Schöll M, Lockhart SN, Schonhaut DR, O'Neil JP, Janabi M, Ossenkoppele R, Baker SL, Vogel JW, Faria J, Schwimmer HD, Rabinovici GD, Jagust WJ (2016). PET imaging of tau deposition in the aging human brain. Neuron.

[40] Schwarz AJ, Yu P, Miller BB, Shcherbinin S, Dickson J, Navitsky M, Joshi AD, Devous MD Sr, Mintun MS (2016) Regional profiles of the candidate tau PET ligand ^18^F-AV-1451 recapitulate key features of Braak histopathological stages. Brain 139: 1539–1550. 10.1093/brain/aww023 10.1093/brain/aww023 26936940

[41] Smith R, Puschmann A, Schöll M, Ohlsson T, van Swieten J, Honer M, Englund E, Hansson O (2016). ^18^F-AV-1451 tau PET imaging correlates strongly with tau neuropathology in MAPT mutation carriers. Brain.

[42] Spina S, Schonhaut DR, Boeve BF, Seeley WW, Ossenkoppele R, O'Neil JP, Lazaris A, Rosen HJ, Boxer AL, Perry DC, Miller BL, Dickson DW, Parisi JE, Jagust WJ, Murray ME, Rabinovici GD (2017). Frontotemporal dementia with the V337M MAPT mutation: tau-PET and pathology correlations. Neurology.

[43] Szardenings AK, Kolb HC, Walsh JC, Chen G, Gangadharmath UB, Kasi D, Liu C, Sinha A, Wang E, Yu C, Zhang W, Chen K, Mocharla VP, Scott PJH (2012) Imaging agents for detecting neurological dysfunction. Patent Application US20120302755 A1 (Siemens Medical Solutions USA, Inc.)

[44] Thal DR, Rueb U, Orantes M, Braak H (2000). Phases of a beta-deposition in the human brain and its relevance for the development of AD. Neurology.

[45] Uchihara T, Nakamura A, Yamazaki M (2001). Mori O (2001) evolution from pretangle neurons to neurofibrillary tangles monitored by thiazin red combined with Gallyas method and double immunofluorescence. Acta Neuropathol.

[46] Vermeiren C, Motte P, Viot D, Mairet-Coello G, Courade JP, Citron M, Mercier J, Hannestad J, Gillard M (2018). The tau positron-emission tomography tracer AV-1451 binds with similar affinities to tau fibrils and monoamine oxidases. Mov Disord.

[47] Vettermann F, Brendel M, Schaenecker S, Haeglinger G, Danek A, Levin J, Bartenstein P, Okamura N, Rominger A (2016) [^18^F]THK-5351 PET in patients with clinically diagnosed progressive supranuclear palsy. J Nucl Med 57 (supplement 2): 457

[48] Wang Y, Mandelkow E (2016). Tau in physiology and pathology. Nat Rev Neurosci.

[49] Xia CF, Arteaga J, Chen G, Gangadharmath U, Gomez LF, Kasi D, Lam C, Liang Q, Liu C, Mocharla VP, Mu F, Sinha A, Su H, Szardenings AK, Walsh JC, Wang E, Yu C, Zhang W, Zhao T, Kolb HC (2013). [(18)F]T807, a novel tau positron emission tomography imaging agent for Alzheimer's disease. Alzheimers Dement.

[50] Zhang W, Arteaga J, Cashion DK, Chen G, Gangadharmath U, Gomez LF, Kasi D, Lam C, Liang Q, Liu C, Mocharla VP, Mu F, Sinha A, Szardenings AK, Wang E, Walsh JC, Xia C, Yu C, Zhao T, Kolb HC (2012). A highly selective and specific PET tracer for imaging of tau pathologies. J Alzheimers Dis.

[51] Zhao Z, Sagare AP, Ma Q, Halliday MR, Kong P, Kisler K, Winkler EA, Ramanathan A, Kanekiyo T, Bu G, Owens NC, Rege SV, Si G, Ahuja A, Zhu D, Miller CA, Schneider JA, Maeda M, Maeda T, Sugawara T, Ichida JK, Zlokovic BV (2015). Central role for PICALM in amyloid-β blood-brain barrier transcytosis and clearance. Nat Neurosci.

