# Supplementary material for: Large inter- and intra-case variability of first generation tau PET ligand binding in neurodegenerative dementias
Source: Acta Neuropathol Commun. 2018 May 1;6:34. doi: 10.1186/s40478-018-0535-z (PMC5928586; doi:10.1186/s40478-018-0535-z)
Supplement: Supplementary file 1 — Table S1. Extended demographic data of cases included in the study. Figure S1. Tau immunohistochemistry in cases with primary tauopathies. Figure S2. Lack of fluorescent tau tracer binding in cases with primary tauopathies. Figure S3. Lack of tau tracer binding to the medial temporal lobe of control cases CTRL1–CTRL4 with and without tau pathology. Figure S4. Representative immunofluorescence images showing AT8 immunoreactive tau inclusions depicted by fluorescent tau tracers. Figure S5. Concomitant labelling of tissue from Alzheimer’s disease cases with fluorescent tau tracers and phospho-tau specific antibodies. Figure S6. Quantitative phosphorimaging with [18F]THK-5117. (PDF 2092 kb) [file 40478_2018_535_MOESM1_ESM.pdf]

Large inter- and intra-case variability of first generation tau PET ligand binding in neurodegenerative dementias

Melissa C. Wren,[1,2] Tammaryn Lashley,[2] Erik Årstad[1] and Kerstin Sander[1,3]

1. Institute of Nuclear Medicine and Department of Chemistry, University College London
2. Institute of Neurology, Department of Molecular Neuroscience, Queen Square Brain Bank for Neurological Disorders, University College London
3. University College London, Radiochemistry, Kathleen Lonsdale Building, 5 Gower Place, London WC1E 6BS, UK; email: k.sander@ucl.ac.uk

Supplementary Material

Additional File	Caption	Page
Table S1	Extended demographic data of cases included in the study	S2
Figure S1	Tau immunohistochemistry in cases with primary tauopathies	S4
Figure S2	Lack of fluorescent tau tracer binding in cases with primary tauopathies	S5
Figure S3	Lack of tau tracer binding to the medial temporal lobe of control cases CTRL1–CTRL4 with and without tau pathology	S7
Figure S4	Representative immunofluorescence images showing AT8 immunoreactive tau inclusions depicted by fluorescent tau tracers	S9
Figure S5	Concomitant labelling of tissue from Alzheimer's disease cases with fluorescent tau tracers and phospho-tau specific antibodies	S10
Figure S6	Quantitative phosphorimaging with [^{18}F]THK-5117	S11

Additional File 1: Table S1 Extended Demographic Data of Cases Included in the Study

Case	Brain Weight (g)	PM Delay (h:min)	Cause of Death	Time in Freezer (y)	Thal Phase	Braak Stage	CERAD	ABC Score	CAA	Synuclein	TDP
**CTRL1**	1330	45:05	Breast carcinoma; cardiac arrest	12	0	0	0	A0B0C0	none	none	none
**CTRL2**	1480	38:50	Mesothelioma	13	0	I	0	A0B1C0	none	none	none
**CTRL3**	1242	49:10	Pancreatic carcinoma	7	0	II	0	A0B1C0	none	none	none
**CTRL4**	1448	38:29	Bronchopneumonia	5	3	III	sparse	A2B2C1	none	none	none
**AD1**	906	92:47	End stage dementia	8	5	VI	frequent	A3B3C3	moderate	none	none
**AD2**	1269	31:10	End stage dementia	5	5	VI	frequent	A3B3C3	mild	none	limbic
**AD3**	1234	73:45	End stage dementia	4	5	VI	frequent	A3B3C3	mild	amygdala	none
**AD4**	1022	33:26	Bronchopneumonia	5	5	VI	frequent	A3B3C3	mild	diffuse neocortical	limbic
**FTDP1**[a)]	1208	60:45	Bronchopneumonia	11	0	0	0	A0B0C0	none	none	none

Additional File 1 : Table S1, continued Extended Demographic Data of Cases Included in the Study

Case	Brain Weight (g)	PM Delay (h:min)	Cause of Death	Time in Freezer (y)	Thal Phase	Braak Stage	CERAD	ABC Score	CAA	Synuclein	TDP
**FTDP2[b)]**	1048	125:00	Sepsis	9	2	II	sparse	A1B1C1	moderate	none	none
**FTDP3[c)]**	1399	58:10	Bronchopneumonia	10	0	0	0	A0B0C0	none	none	none
**PICK1**	1166	24:00	Unknown	6	1	III	sparse	A1B2C1	none	none	none
**PICK2**	1040	43:30	End stage dementia	6	3	0	sparse	A2B0C1	none	none	none
**PSP1**	1580	80:20	End stage dementia	12	2	0	sparse	A1B0C1	none	none	none
**PSP2**	1177	36:50	End stage dementia	5	1	0	sparse	A1B0C1	none	none	none
**CBD**	1100	80:48	Multiple organ failure	10	0	0	0	A0B0C0	none	none	none

Abbreviations: CTRL, control; AD, Alzheimer's disease; FTDP, frontotemporal dementia with parkinsonism linked to chromosome 17; PICK, Pick's disease; PSP, progressive supranuclear palsy; CBD, corticobasal degeneration; PM, *post-mortem*; CAA, cerebral amyloid angiopathy; TDP, TAR DNA-binding protein 43.

Genetic variants: a) MAPT R406W; b) MAPT Δ280K; c) MAPT 10+16.

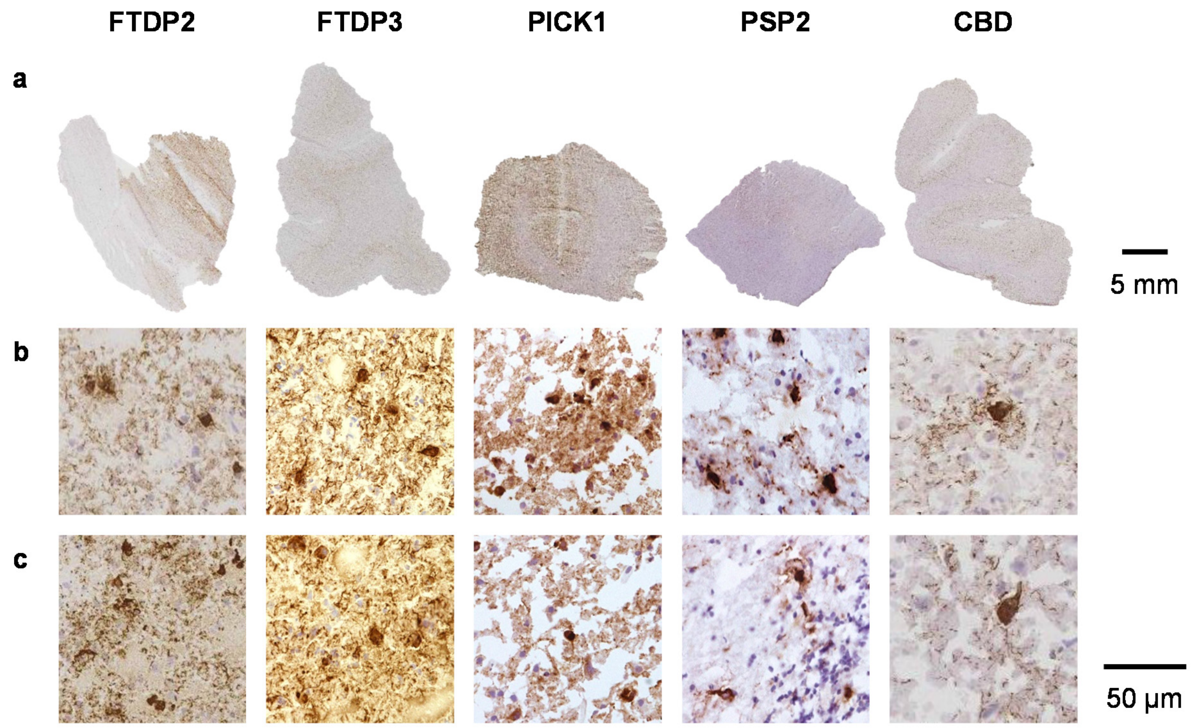

Additional file 1 : Figure S1 Tau immunohistochemistry in cases with primary tauopathies

a) Representative images of whole brain sections from the frontal cortex showing macroscopic AT8 distribution. **b)** Typical AT8 positive tau aggregates in frontal cortex. **c)** Typical AT8 positive tau aggregates in temporal cortex (FTDP, PICK), cerebellum (PSP) and parietal cortex (CBD).

Abbreviations: FTDP, frontotemporal dementia and parkinsonism linked to chromosome 17; PICK, Pick's disease; PSP, progressive supranuclear palsy; CBD, corticobasal degeneration.

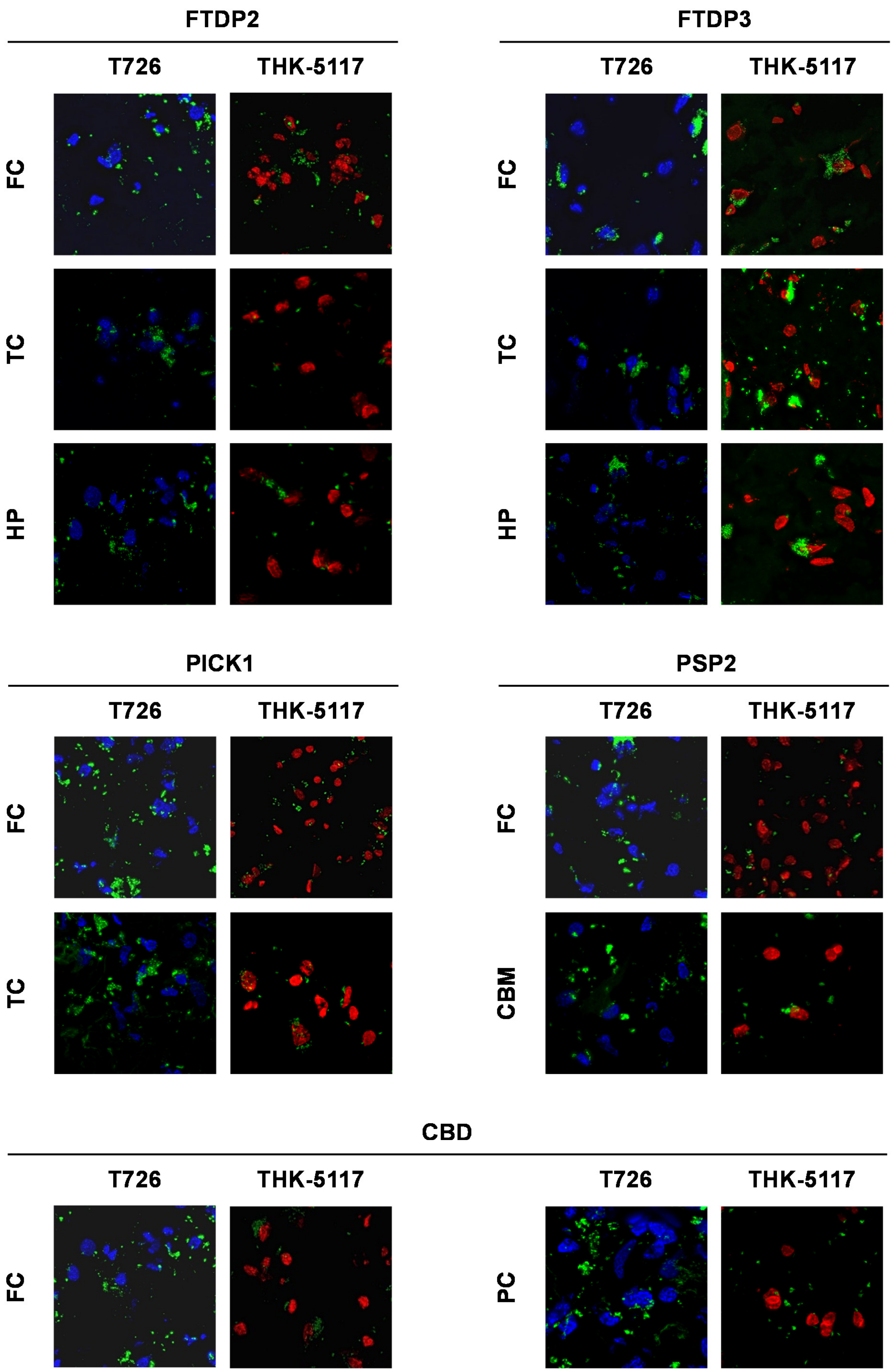
FTDP2
T726
THK-5117
FC
TC
HP
FTDP3
T726
THK-5117
FC
TC
HP
PICK1
T726
THK-5117
FC
TC
PSP2
T726
THK-5117
FC
CBM
CBD
T726
THK-5117
FC
T726
THK-5117
PC

on page S5:

Additional file 1 : Figure S2 Lack of fluorescent tau tracer binding in cases with primary tauopathies

Absence of T726 (co-stained with DAPI in blue) and THK-5117 (co-stained with Nissl Neurotrace 640 in red) binding to pathological structures in primary tauopathies. The green signal results from autofluorescence of lipofuscin pigment granules.

Abbreviations: FTDP, frontotemporal dementia and parkinsonism linked to chromosome 17; PICK, Pick's disease; PSP, progressive supranuclear palsy; CBD, corticobasal degeneration; FC, frontal cortex; TC, temporal cortex; PC, parietal cortex; CBM, cerebellum.

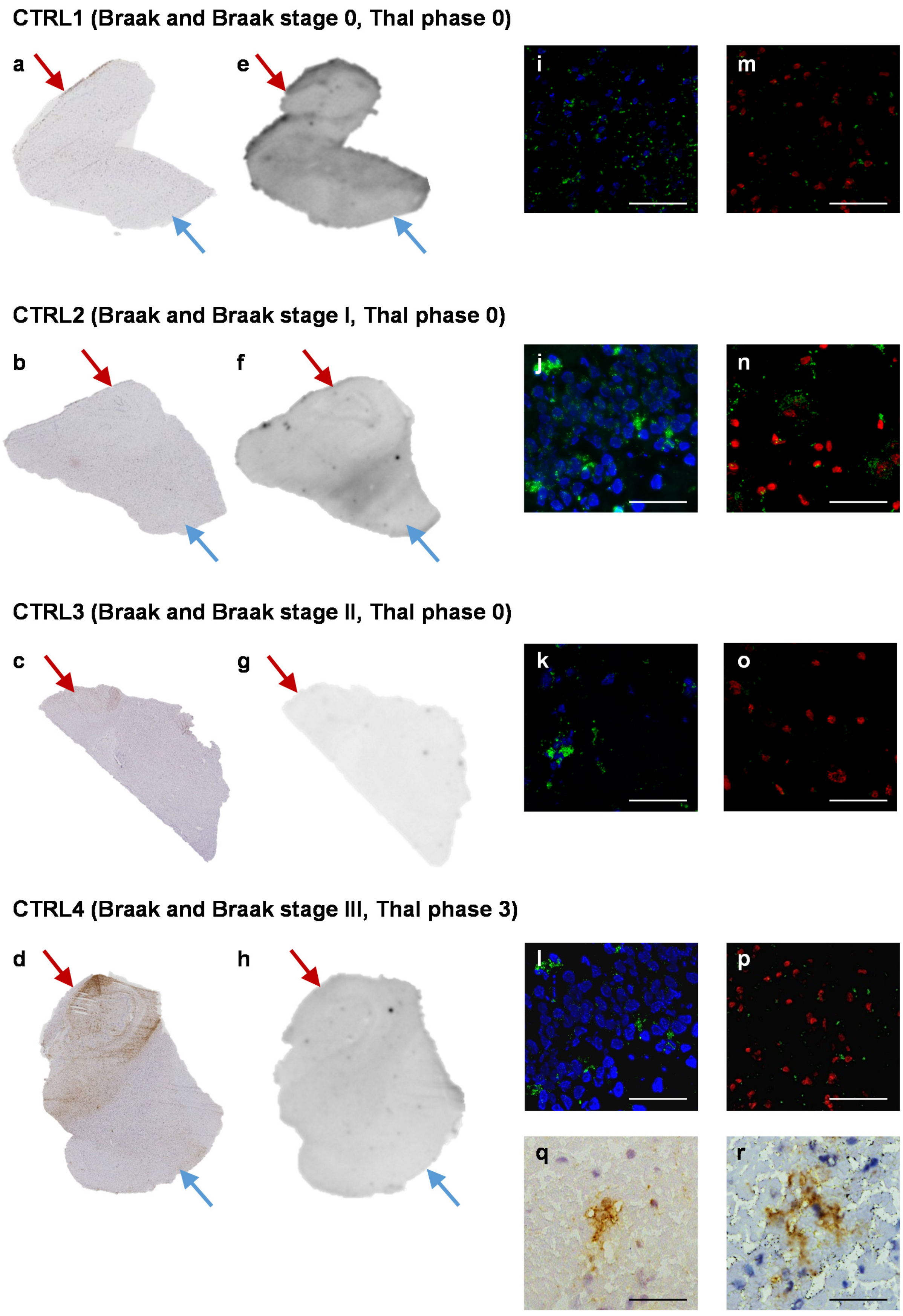
CTRL1 (Braak and Braak stage 0, Thal phase 0)
a
e
i
m
CTRL2 (Braak and Braak stage I, Thal phase 0)
b
f
j
n
CTRL3 (Braak and Braak stage II, Thal phase 0)
c
g
k
o
CTRL4 (Braak and Braak stage III, Thal phase 3)
d
h
l
p
q
r

on page S7:

Additional file 1 : Figure S3 Lack of tau tracer binding to the medial temporal lobe of control cases CTRL1–CTRL4 with and without tau pathology

a–d) Tau load in hippocampus (red arrows) and entorhinal cortex (blue arrows) as determined by immunohistochemistry with AT8. **e–h)** Phosphorimages showing lack of [^{18}F]THK-5117 binding to hippocampus (red arrows) and entorhinal cortex (blue arrows), respectively. **i–l)** Absence of T726 binding to pathological structures; T726 in green, DAPI in blue; scale bar insets 50 µm. **m–p)** Absence of THK-5117 binding to pathological structures; THK-5117 in green, Nissl Neurotrace 640 in red; scale bar insets 50 µm. **i–p)** The green signal pertains to autofluorescence of lipofuscin pigment granules. **q/r)** Absence of radiation triggered silver aggregation after nuclear emulsion autoradiography and subsequent AT8 immunohistochemistry; AT8 in brown; scale bar insets 50 µm.

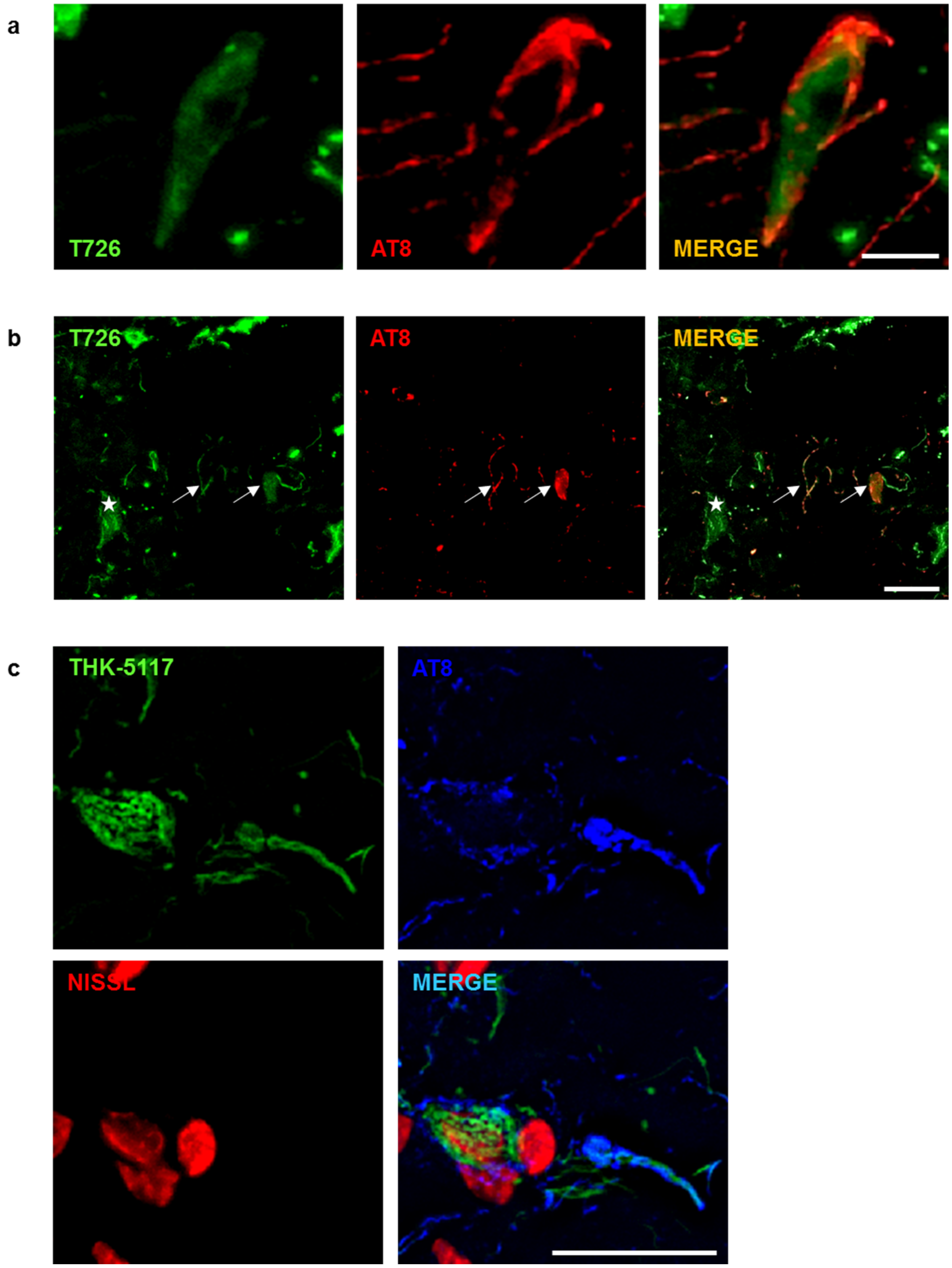

Additional file 1 : Figure S4 Representative immunofluorescence images showing AT8 immunoreactive tau inclusions depicted by fluorescent tau tracers

a) T726 labelled neurofibrillary tangle (T726 in green; AT8 filament immunoreactivity in red; scale bar 10 µm). **b)** T726 labelled pre-tangles (T726 in green; AT8 filament immunoreactivity in red; scale bar 25 µm. **c)** THK-5117 labelled neurofibrillary tangle (THK-5117 in green; AT8 filament immunoreactivity in red; DAPI in blue; scale bar 20 µm).

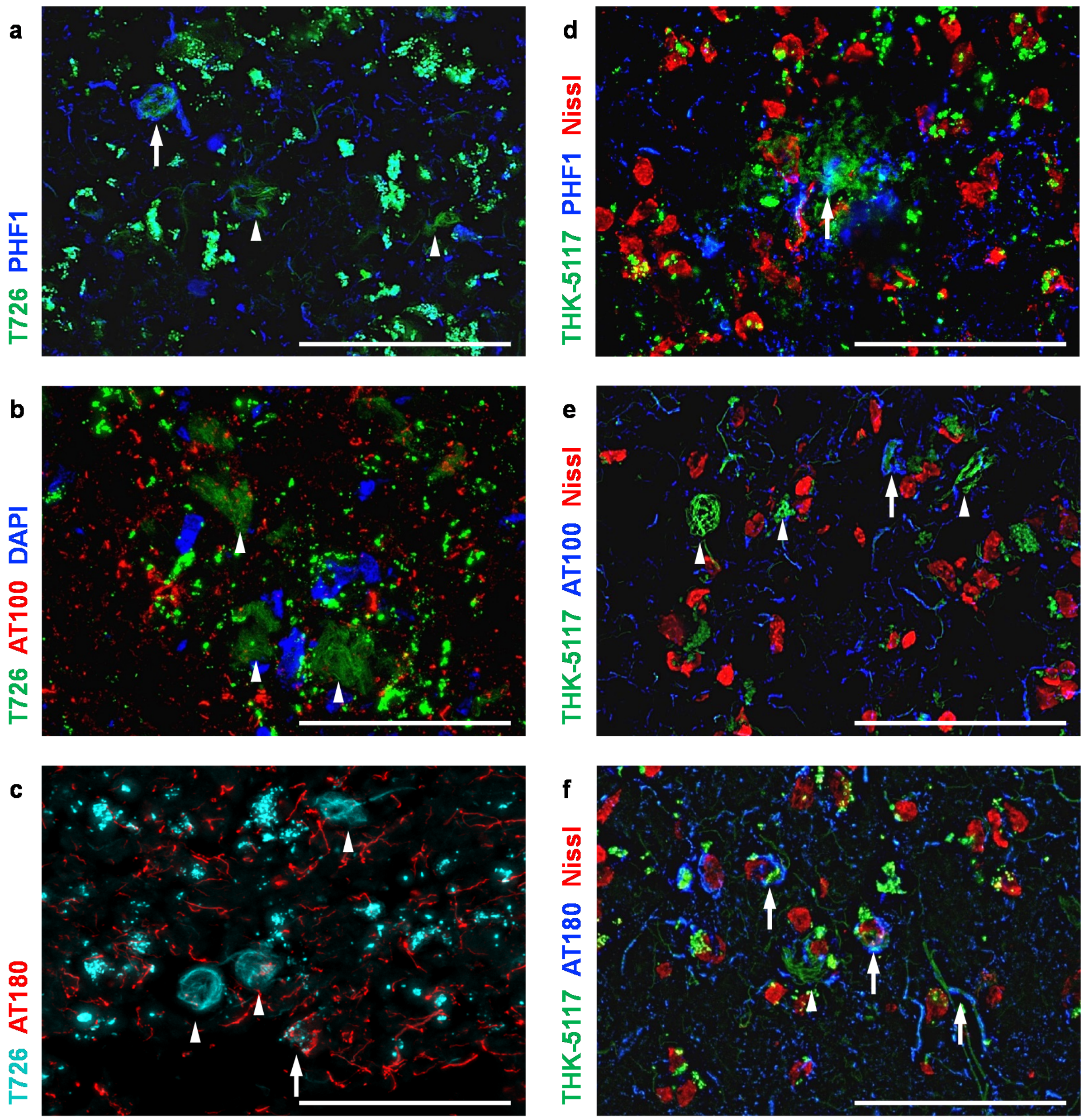

Additional file 1 : Figure S5 Concomitant labelling of tissue from Alzheimer's disease cases with fluorescent tau tracers and phospho-tau specific antibodies

Representative images showing tau tracer labelled structures negative (arrow heads) and positive (arrows) for antibody binding; scale bar insets 50 µm. **a)** T726 in green, PHF1 in blue. **b)** T726 in green, AT100 in red, DAPI in blue. c) T726 in turquoise, AT180 in red. **d)** THK-5117 in green, PHF1 in blue, Nissl Neurotrace 640 in red. e) THK-5117 in green, AT100 in blue, Nissl Neurotrace 640 in red. f) THK-5117 in green, AT180 in blue, Nissl Neurotrace 640 in red.

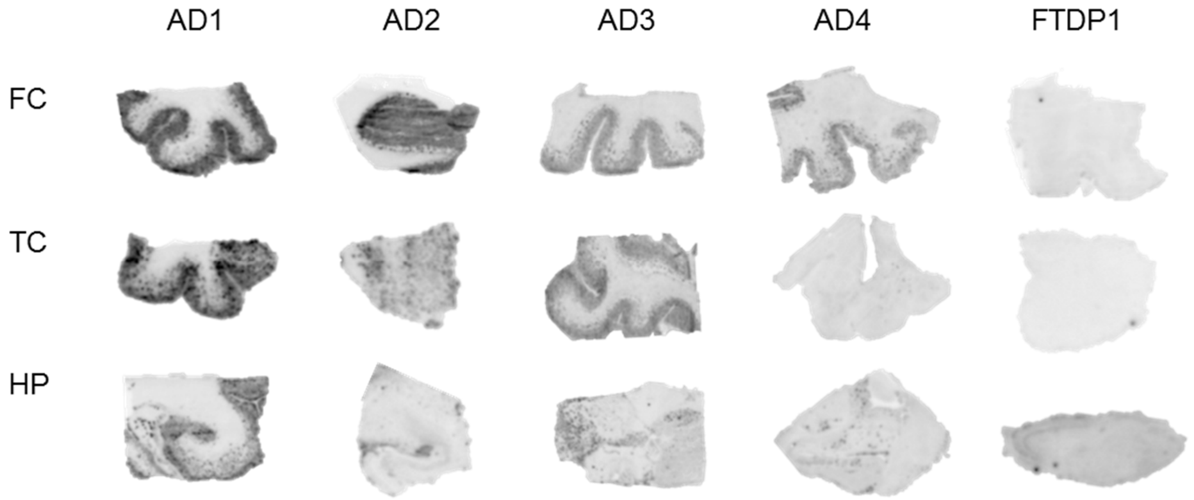

Additional file 1 : Figure S6 Quantitative phosphorimaging with [^{18}F]THK-5117

Representative phosphorimages of total tracer binding to the Alzheimer’s disease cases AD1–AD4, and the FTDP-17 case FTDP1.

Abbreviations: FC, frontal cortex; TC, temporal cortex; HP, hippocampus.
